# A novel IVN-entropy based distance-driven MARCOS framework for evaluating and ranking global green hydrogen-producing countries

**DOI:** 10.1186/s12859-026-06425-z

**Published:** 2026-04-16

**Authors:** Venkata Prasanna Nagari, Vinoth Subbiah

**Affiliations:** Department of Mathematics and Statistics, Vignan’s Foundation for Science, Technology and Research, Vadlamudi, Andhra Pradesh 522213 India

**Keywords:** Green hydrogen, Multi-criteria decision-making, Interval-valued neutrosophic sets, Entropy weighting, Distance-based MARCOS, Sustainability assessment

## Abstract

**Background:**

The global transition toward carbon neutrality has intensified the need for reliable and data-driven methods to evaluate green hydrogen production across countries. Existing approaches to multi-criteria decision-making (MCDM) under uncertainty often struggle with the simultaneous treatment of data ambiguity, indeterminacy, and conflicting information. To address these limitations, this study develops a hybrid interval-valued neutrosophic (IVN) framework that combines entropy-based objective weighting with a distance-driven MARCOS (Measurement of Alternatives and Ranking according to Compromise Solution) model.

**Results:**

The proposed IVN-entropy-NC-SIVNDM MARCOS framework introduces a new normalized centroid-spread IVN distance measure (NC-SIVNDM) that captures the uncertainty and hesitancy inherent in expert or data-derived evaluations. Eight quantitative indicators such as covering production capacity, electrolyser deployment, investment maturity, cost efficiency, and energy intensity were used to assess ten leading green hydrogen-producing countries based on data from the international energy agency (IEA-global hydrogen review 2024). Objective entropy-based weights revealed that current low-emissions hydrogen production and installed electrolyser capacity were the most influential criteria. The proposed model produced consistent and discriminative country rankings, with sensitivity and comparative analyses confirming its robustness and reliability against perturbations in criteria weights.

**Conclusions:**

The IVN-Entropy-NC-SIVNDM MARCOS model provides a transparent, replicable, and computationally efficient decision-support tool for benchmarking national performance in green hydrogen development. By integrating objective entropy weighting and a novel distance-based neutrosophic measure, the framework enhances ranking stability and interpretability under uncertainty. This methodology offers a valuable contribution to sustainable energy planning and may assist policymakers, investors, and researchers in guiding the global shift toward a green hydrogen economy and net-zero emission goals.

## Background

The global energy system faces significant challenges related to carbon emissions, primarily due to ongoing dependence on fossil fuels for industrial production, transportation, and power generation. In 2023, hydrogen production from fossil fuels resulted in approximately 920 million tonnes of CO_2_ emissions, a figure comparable to the total annual emissions of countries like Indonesia and France (IEA, 2024). These emissions substantially intensify climate change, leading to higher global temperatures, more extreme weather, and biodiversity loss. Despite global initiatives aimed at decarbonization, the predominant method of hydrogen production remains reliant on unabated natural gas and coal, resulting in significant carbon intensity in critical sectors such as refining, ammonia, methanol, and steel production. The international energy agency states that attaining net-zero emissions by 2050 necessitates the substitution of conventional hydrogen with low-emission alternatives, especially green hydrogen generated via renewable-powered electrolysis [1].

Green hydrogen is essential for decreasing reliance on fossil fuels and facilitating significant decarbonization in challenging sectors, such as heavy industry and long-distance transportation. It serves as an efficient energy carrier, capable of storing excess renewable electricity and improving the flexibility of energy systems. In light of global net-zero commitments, green hydrogen emerges as one of the limited scalable solutions for achieving zero carbon emissions in industrial transformation. Thus, expediting its implementation is crucial for addressing climate change and facilitating a sustainable energy transition [2].

Green hydrogen is hydrogen produced by water electrolysis using renewable energy sources such as solar, wind, or hydropower. Because the process is entirely powered by clean electricity, it eliminates direct carbon emissions and is widely regarded as the cleanest form of hydrogen [3]. Unlike gray or blue hydrogen, which depend on fossil fuels or carbon capture technologies, green hydrogen offers a fully sustainable pathway for producing energy carriers applicable across power generation, industrial processes, and transportation systems [4]. Although current challenges related to production costs and infrastructure remain, green hydrogen is expected to play a pivotal role in achieving net-zero greenhouse gas emissions and advancing the sustainable development goals by mid-century [5].

The integration of renewable energy-based hydrogen systems is increasingly recognized as a transformative strategy for decarbonizing energy-intensive sectors while improving grid flexibility and resilience [6]. However, the absence of unified global standards highlights the need for precise terminology, certification mechanisms, and guarantees of origin to ensure transparency and equitable trade [7]. Regions with abundant renewable resources, such as the middle east and north Africa, exhibit strong potential for green hydrogen production. For instance, Saudi Arabia’s vast solar and wind resources position it as a prospective global leader in green hydrogen exports, supporting both emission reduction targets and economic diversification goals [8].

Despite growing interest and policy support, the global implementation of green hydrogen remains limited. While integrated hybrid energy systems can significantly reduce CO₂ emissions and improve the utilization of renewable energy [9], challenges related to policy uncertainty, investment risks, and regulatory transparency persist, particularly in emerging hydrogen economies [10]. Moreover, only a small fraction of announced green hydrogen projects have reached operational status, revealing a considerable gap between ambition and execution [11]. Economic incentives and international trade mechanisms have the potential to substantially reduce production costs, but their effectiveness depends on coordinated policy frameworks and technological progress [12].

The multifaceted nature of green hydrogen development necessitates decision-support tools that can concurrently assess economic, environmental, technological, and policy-related aspects. Multi-criteria decision-making (MCDM) methodologies have consequently gained prominence in sustainability evaluations [13–15]. The MARCOS (Measurement of Alternatives and Ranking according to Compromise Solution) approach is esteemed for its computational efficiency and consistent ranking performance. Nonetheless, its utilization in highly uncertain contexts, especially those marked by data ambiguity and indeterminacy, remains constrained.

Research and production of green hydrogen are increasingly acknowledged as vital for improving the sustainability of the global energy system, offering significant potential for profound decarbonization and economic diversification. Leveraging substantial solar and wind resources in nations like Algeria, Saudi Arabia, and Jordan can markedly diminish emissions while enhancing energy reliability [16]. Improvements in electrolyser efficiency and the adoption of renewable energy have enhanced the technical viability of large-scale hydrogen production. However, obstacles persist regarding infrastructure, production costs, and water access [17]. The creation of strong international standards and certification systems is essential for enhancing market transparency and enabling sustainable trade. Evidence suggests that robust legislative frameworks, financial incentives, and regional collaboration are essential for realizing the complete potential of hydrogen as a sustainable energy carrier [18].

Recent studies emphasize the significance of green hydrogen in facilitating deep decarbonization and ensuring grid stability via the integration of renewable energy sources. Analyses of techno-economic indicators indicate that optimally designed off-grid hybrid solar–wind systems, exemplified by models from Australia, can achieve competitive hydrogen production costs [19]. Stochastic optimization frameworks indicate that hydrogen storage, specifically long-term liquid storage, can improve the stability of renewable energy grids [20]. Furthermore, research on offshore systems indicates that integrating hydropneumatic storage with renewable energy sources, such as wind power, enhances production reliability and consistency [21]. These findings indicate that integrating advanced modeling, renewable energy synergies, and supportive governance can expedite the establishment of a sustainable hydrogen economy.

Despite the progress in hydrogen-related modeling and policy studies, very few attempts have integrated MCDM frameworks within an IVN environment to objectively evaluate national performance in green hydrogen development. IVN logic, first introduced by Smarandache (1995), represents uncertain information using three membership intervals: truth, indeterminacy, and falsity. This tri-parametric structure effectively captures vagueness and hesitation in expert or empirical data, thereby improving ranking stability under uncertainty.

Recent studies indicate that green hydrogen is essential for global decarbonization; however, its large-scale production faces significant technological and economic challenges. Significant challenges include substantial energy consumption and increased manufacturing costs, largely due to the high cost of electrolysers and the fluctuating output of renewable energy sources. Material scarcity, driven by dependence on metals such as platinum and iridium, and restricted water availability in remote areas further limit environmentally sustainable production. Furthermore, insufficient infrastructure for logistics, transportation, and resource distribution, along with fragmented regulations and variable financial incentives, continue to obstruct deployment. Advancements in electrolyser technology, especially through the integration with renewable energy and coordinated policy measures, indicate that GH₂ may attain commercial viability and environmentally sustainable operation by the mid-twenty-first century [22, 23].

The major contributions of the study are outlined below.

1. A novel IVN-Entropy-NC-SIVNDM MARCOS framework is developed by integrating entropy-based objective weighting with distance-driven evaluation to improve decision accuracy and robustness.
2. A new normalized centroid-spread IVN distance (NC-SIVND) measure is introduced to effectively capture uncertainty, indeterminacy, and expert hesitancy.
3. The NC-SIVNDM MARCOS model extends the classical MARCOS approach, enhancing discrimination ability and ranking consistency under uncertain conditions.
4. The IVN-entropy weighting method ensures objective and bias-free assessment of criterion importance without subjective influence.
5. Sensitivity and comparative analyses confirm the stability and reliability of the proposed model across different weighting scenarios.
6. The framework provides a transparent, generalizable decision-support tool for benchmarking green hydrogen-producing countries and supporting sustainable policy development.

This research is organized as follows. "Background" section presents the background, objectives, and significance of the study. "Literature review" section describes the materials and methods used to construct the framework. "Methods" section details the proposed methodology, including the development and integration of the models. "Results" section discusses the results and illustrates the application of the framework to real-world scenarios. "Discussion" section provides an in-depth analysis and interpretation of the findings, situating them within the context of previous research and highlighting practical implications. Finally, "Conclusions" section summarizes the key findings and offers recommendations for future research directions.

The proposed IVN-Entropy–NC-SIVNDM MARCOS framework distinguishes itself from previous MCDM-based benchmarking studies, which mainly focus on enhancing ranking accuracy or stability. It offers more profound policy-relevant insights by explicitly identifying structural factors, such as existing low-emission hydrogen production and electrolyser deployment, that significantly influence national competitiveness. This allows policymakers to transcend ordinal rankings and comprehend the fundamental performance disparities that affect global leadership in green hydrogen development.

The uncertainty associated with national-level green hydrogen evaluation encompasses not only ambiguity or randomness but also a significant level of indeterminacy. This indeterminacy stems from incomplete project execution, delays between announced and actual capacities, varying reporting standards among countries, and uncertainty concerning the effects of policy implementation. Traditional fuzzy and intuitionistic fuzzy frameworks predominantly represent ambiguity via membership and non-membership functions; yet, they tacitly presuppose that the knowledge at hand is comprehensive and definitive. Conversely, interval-valued neutrosophic (IVN) theory distinctly characterizes indeterminacy as a separate dimension, facilitating a more accurate depiction of partially known or developing energy system data.

## Literature review

Single-criterion methodologies are insufficient for accurate decision-making because sophisticated energy infrastructures often involve many contradictory and interconnected criteria. MCDM methods incorporate quantitative and qualitative considerations to prioritize alternatives transparently and objectively. These strategies balance technological, economic, environmental, and social goals to support sustainable energy policies [13–15]. MCDM evaluates production cost, efficiency, carbon intensity, and resource availability in green hydrogen production. This approach helps nations and policymakers choose the best hydrogen technology scaling plans in the face of uncertainty [24]. MCDM also supports fair benchmarking, investment prioritization, and policymaking to accelerate global decarbonization [25].

Entropy-based weighting has become important because it can objectively assess the importance of criteria from data, thereby removing subjective bias and enhancing decision reliability. The integration of objective weighting with robust ranking procedures, such as MARCOS, improves model validity, transparency, and reproducibility. This research presents a framework for evaluating and ranking countries based on their performance in green hydrogen production. It uses the IVN entropy method for objective-criterion weighting and the NC-SIVNDM MARCOS model for distance-driven ranking. This study includes two primary research areas: assessing the performance of global green hydrogen generation and developing MCDM methods that address uncertainty, indeterminacy, and data complexity in sustainability evaluations.

## Research investigations on the analysis of the sustainability of green hydrogen production

Recent studies have increasingly used MCDM approaches to evaluate and enhance green hydrogen (GH₂) systems across diverse geographical and technological settings. A GIS-AHP methodology identified suitable sites for solar-based hydrogen generation in southern Thailand, accounting for environmental, technological, and economic factors [26]. A multicriteria analysis of seawater electrolysis technologies identified proton exchange membrane electrolysis as the optimal method for offshore hydrogen production [27]. The best–worst method (BWM) assessed GH_2_ supply chain risks in Europe's hard-to-abate sectors, pinpointing substantial capital expenditures and insufficient policy frameworks as primary impediments [28]. The VIKOR and TOPSIS approaches enabled effective decision modeling for GH₂ development in Greece. Integrated MCDM frameworks continue to guide sustainable site selection and technology prioritization in renewable hydrogen systems [29].

Subsequent research has investigated the suitability of GH₂ in Tunisia and North Africa through GIS-AHP, integrating technoeconomic, social, and geopolitical factors [30]. In Spain, GIS-AHP assessed the solar photovoltaic potential for industrial hydrogen production, highlighting the significance of proximity to infrastructure [31]. A hybrid entropy-MARCOS framework improved offshore GH₂ systems by evaluating technoeconomic, safety, and environmental risks [32]. A SWOT-MCDM model delineated strategic pathways for Tunisia's GH₂ transition, emphasizing renewable potential and regional collaboration [33]. Complementary technoeconomic-TOPSIS analyses optimized hybrid renewable systems incorporating hydrogen storage for off-grid applications, illustrating cost-effective and low-emission designs [34].

A revised EDAS framework, integrating both qualitative and quantitative criteria, identified wind electrolysis as the most sustainable technique for GH_2_ production [35]. Similarly, an entropy-weighted TOPSIS model determined wind-alkaline electrolysis as the most feasible renewable-electrolyser configuration [36]. In Malaysia, the AHP method supported industrial decarbonization by highlighting large-scale solar-based electrolysis as the most advantageous GH₂ source. In Turkey, the heuristic CELO_GH algorithm optimized facility locations by considering renewable energy potential, logistics, and economic viability [37]. A comparative MCDM analysis using TOPSIS, ARAS, SAW, and CODAS indicated that Ceará, Brazil, offers excellent conditions for green hydrogen generation based on renewable resources and infrastructure [38].

Recent research has used fuzzy and neutrosophic MCDM models to address uncertainty in the evaluation of green hydrogen. Models based on blockchain interval-valued fuzzy SWARA-EDAS have identified economic and technological risks as significant challenges in supply chains, while also enhancing transparency and robustness [39]. A neutrosophic framework applied to wind-based hydrogen production in Pakistan demonstrated greater efficacy in handling indeterminate data than traditional methods [40].

## Research gap and contributions of the present study

The present research offers a novel integrated model that analyses and ranks countries on the basis of green hydrogen production outcomes via the IVN-Entropy approach for objective criterion weights and the NC-SIVNDM MARCOS procedure for ranking alternatives. Criteria weights are critical in MCDM because they affect the results. The weighting method can be subjective, objective, or combined [41]. Subjective weighting relies on expert opinions and decision-makers’ judgments about the relative relevance of criteria, but with limited or inconsistent expert input, it can be time-consuming and biased [42]. However, objective weighting approaches mathematically calculate weights from the data, reducing subjective bias and improving computational performance. Objective methods yield more accurate, data-driven results. Typical objective methods encompass the mean weight, standard deviation, statistical variance, and entropy-based schemes [43].

Entropy, initially introduced by Shannon in 1948, measures the information embedded in decision data and provides an objective assessment of the significance of each criterion. A variety of studies have developed entropy-based frameworks in interval-valued neutrosophic (IVN) settings to enhance the management of uncertainty and improve decision analysis accuracy. For example, various similarities, inclusion, and dissimilarity measures have been introduced, demonstrating that entropy can significantly enhance robustness and interpretability in IVN-based MCDM models [44–48]. These developments collectively underscore entropy's ability to yield consistent, data-driven weighting outcomes in the face of uncertainty.

The MARCOS method, developed by Stević et al. [49], is a well-established and highly effective MCDM strategy that evaluates options using utility-based functions. This method addresses the shortcomings of prior models. Traditional, fuzzy, or single-valued neutrosophic settings are used by the majority of existing MARCOS applications. A formulation based on distance, operating within the IVN environment, and incorporating objective weights generated from entropy is still largely under investigation.

For this reason, the present investigation addresses these methodological gaps by proposing an IVN-Entropy-NC-SIVNDM MARCOS model. This model combines objective entropy weighting with a new distance-driven evaluation framework. In addition to efficiently managing uncertainty and indeterminacy, the proposed model improves ranking accuracy, stability, and interpretability. Finally, to confirm the robustness and coherence of the model, detailed sensitivity and comparative analyses are conducted.

The literature evaluated is summarized in Table 1, which also highlights the gaps that currently exist and the unique contributions the current work has made to advancing IVN-based MCDM applications for sustainable green hydrogen evaluation.

**Table 1:** Summary of previous studies related to MARCOS-based MCDM models

Study/year	Environment/framework used	Weighting method	Ranking method	Distance measure	Application area	Key finding/limitation
2020 [50]	Fuzzy environment	Fuzzy PIPRECIA	Fuzzy MARCOS	No	Traffic risk analysis	Introduced fuzzy MARCOS but lacked objective weighting and indeterminacy treatment
2021 [51]	Integrated fuzzy & neutrosophic fuzzy (NF)	Fuzzy FUCOM	NF-MARCOS	No	Sustainable road transportation (Alternative fuel vehicles)	Improved decision reliability; limited by computational complexity and small expert sample
2021 [52]	Intuitionistic-Fuzzy (IF)	Intuitionistic Fuzzy Weighted Averaging	IF-MARCOS	No	Insurance company evaluation (health services)	Effectively handled uncertainty with stable rankings; limited to one-country insurance case
2022 [53]	Interval-valued intuitionistic fuzzy sets (IVIFSs)	IVIF-Shannon Entropy + Similarity Measure	Hybrid IVIF-Extended-VIKOR & IVIF-MARCOS	No	Sustainable supplier selection (healthcare)	Hybrid IVIF-VIKOR-MARCOS improves robustness under uncertainty; complex and few experts
2022 [54]	Bayesian MCDM framework	Bayesian Best–Worst Method	MARCOS	No	Regulatory Risk Assessment for Power Grid Enterprises in China	Enhanced stability and ranking accuracy; limited to one industry and expert sample
2022 [55]	Single-valued neutrosophic set (SVNS)	Combined weighting using BWM and improved CRITIC	MARCOS integrated with Regret Theory	No	Pharmaceutical enterprise performance evaluation	Enhanced decision realism and uncertainty handling; limited by high computation and parameter sensitivity
2023 [56]	Classical	Entropy	MARCOS	No	Third-Party Logistics (3PL) performance evaluation	CO₂ emissions most critical; robust via sensitivity tests; limited to 15 3PLs
2023 [57]	Hybrid (Classical + Fuzzy)	Entropy + Fuzzy BWM	MARCOS	No	Power grid investment (renewable energy)	Integrated objective-subjective weights; identified top-performing project; validated by sensitivity analysis
2023 [58]	Trapezoidal fuzzy–SVN-F	TrF-BWM	SVN-F-MARCOS	No	Surface water treatment plants	Novel hybrid model improving ranking accuracy under uncertainty; first use in this domain
2024 [59]	Fuzzy environment	Fuzzy Analytic Hierarchy Process (AHP)	Fuzzy MARCOS	No	Graphic design sustainability	First hybrid FAHP-FMARCOS model for sustainable design; validated via sensitivity analysis; limited to expert-based evaluation
2024 [60]	Pythagorean fuzzy (PF)	PF-CRITIC	PF-MARCOS	No	Sustainable food supplier selection	Developed first PF-CRITIC-MARCOS for supplier selection; captures intercriteria correlation; robust by sensitivity analysis
2024 [61]	Pythagorean fuzzy (PF)	PF-MEREC	PF-MARCOS	No	Sustainable forest resource management	First application of PF-MEREC-MARCOS for forest management; effectively handles uncertainty; results verified via sensitivity and comparative analysis
2024 [62]	Interval type-2 spherical fuzzy	CI-ITARA	CI-TODIM-MARCOS	No	Sustainable supply chain under risk criteria	Introduction of new IT2SFS-based hybrid MCDM; improved intercriteria interaction; effective under uncertain and risky conditions
2025 [63]	Fuzzy environment	Fuzzy Best–Worst Method (BWM)	Fuzzy MARCOS	No	Failure prioritization in wind–solar hybrid energy systems (HRES)	Prioritizes key failures; stable under analyses; limited by lack of real-world dynamic data
2025 [64]	Fuzzy environment	Combined Fuzzy LBWA & Fuzzy LMAW (hybrid weighting)	MARCOS	No	Performance evaluation of commercial banks (Pakistan case study)	Produces consistent, robust rankings across sensitivity tests; demonstrated on Pakistani banks generalizability needs other contexts
2025 [65]	Refined fuzzy environment	(Fuzzy) Weighting variants tested (Equal, ROC, Rank Sum, Entropy comparisons discussed)	Refined Fuzzy MARCOS (Quasi-D-Overlap aggregation)	No	Sensor selection in IoT-based healthcare (real case)	Enhance discrimination and consistency; manages criterion overlap; validated on healthcare sensors but needs broader testing
2025 [66]	Hybrid neutrosophic environment	SvNCN-PIPRECIA; Objective: Entropy; Combined via Uninorm operator	SPA-MARCOS (dual-driven ranking)	No	Dual-driven evaluation (data-trading platforms/MAGDM applications)	Integrates knowledge- and data-driven inputs for robust decisions; limited by high complexity and computational demand
Proposed Study	Interval-valued neutrosophic (IVN) environment	IVN-Entropy	NC-SIVNDC MARCOS (Distance based)	YES	Evaluation of GH production countries	Introduces a novel IVN Entropy-based distance-driven MARCOS framework that objectively determines criteria weights, effectively models uncertainty and indeterminacy, and validates robustness through sensitivity and comparative analyses

PIPRECIA, Pivot Pairwise Relative Criteria Importance Assessment; FUCOM, Full Consistency Method; CI-ITARA, Choquet integral-indifference threshold-based attribute ratio analysis; F-LBWA, Fuzzy Level-Based Weight Assessment; F-LMAW, Fuzzy Logarithm Methodology of Additive Weights; CRITIC, CRiteria Importance Through Intercriteria Correlation; MEREC, Method based on the Removal Effects of Criteria; NC-SIVNDC-MARCOS, Normalized Centroid-Spread Interval Valued Neutrosophic Distance Measure-Measurement Alternatives and Ranking according to the COmpromise Solution

## Methods

This section formulates a methodological framework. It integrates IVN-Entropy objective weighting and the distance-driven MARCOS model. These tools are used to evaluate and rank green hydrogen-producing countries under uncertainty.

### Research framework

**Fig. 1:**
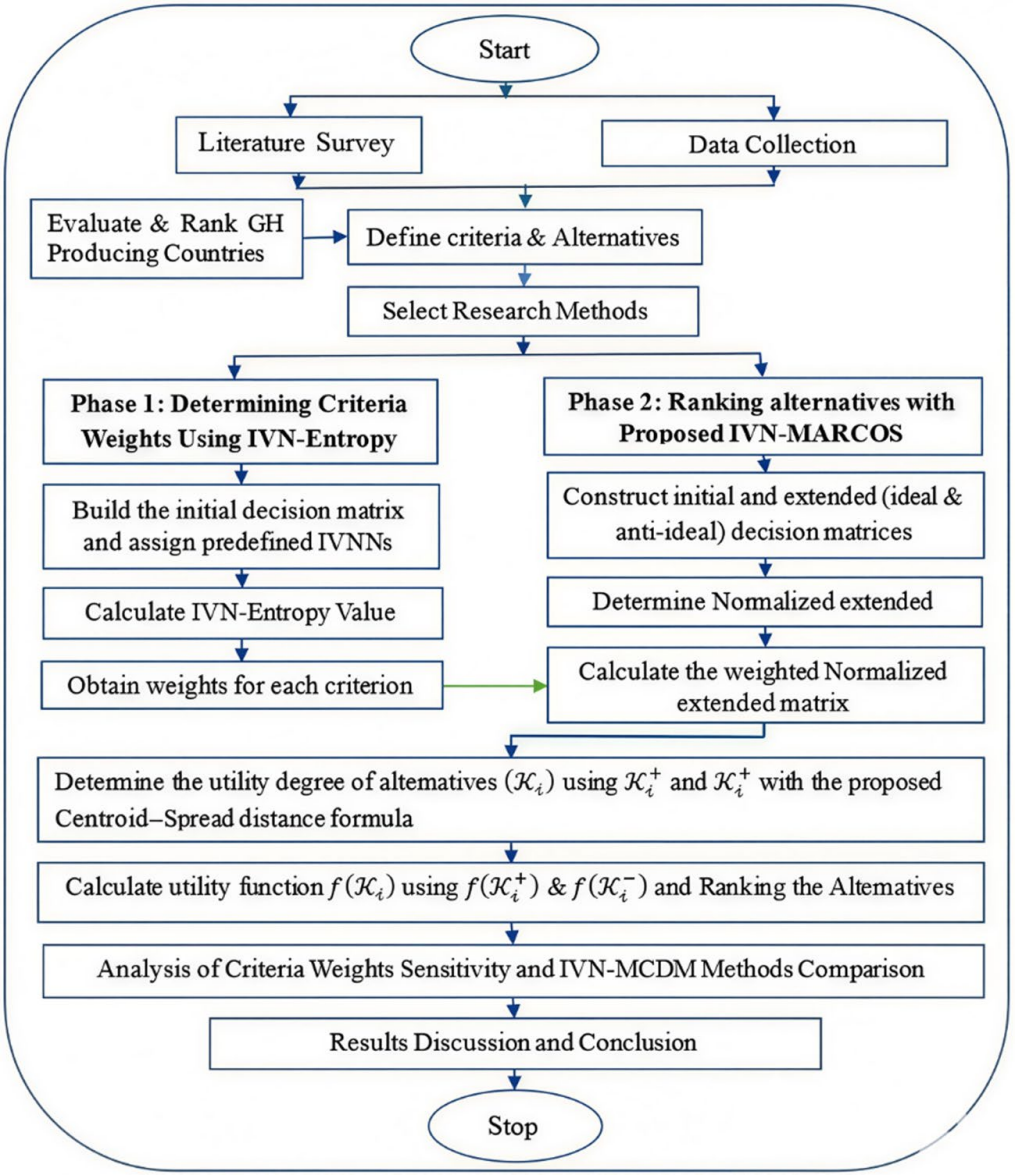
Flowchart of the proposed research methodology

MCDM is widely accepted as an effective technique for addressing complex decision-making challenges by reconciling diverse priorities, risks, and constraints. This study presents a research framework for investigating and ranking countries in green hydrogen production using eight criteria and ten alternatives, as demonstrated in Fig. 1. Initially, the weight coefficients for each criterion are determined using the interval-valued neutrosophic (IVN) entropy method. The proposed NC-SIVNDM MARCOS technique is subsequently used to rank the alternatives. To guarantee the robustness and applicability of the proposed framework, a sensitivity analysis of the criteria weights and a comparative study of different IVN-based MCDM approaches are conducted.

### Preliminaries

#### Definition (Neutrosophic sets)

Neutrosophic set theory was developed by [67] in 1995. Unlike the classical and fuzzy approaches, neutrosophic set theory is an approach that also includes uncertainty points. There are three levels under the headings of truth (Truth-$\mathcal{T}$), uncertainty (Indeterminacy-$\mathcal{I}$), and falsity (Falsity-$\mathcal{F}$) in neutrosophic set $\mathcal{A}$ in the universe $\mathcal{S}$. The neutrosophic set is shown as $\mathcal{A}$ in Eqs. (1) and (3).

1 $$\mathcal{A}=\{\langle \mathcal{x}, \left({\mathcal{T}}_{\mathcal{A}}\left(\mathcal{x}\right), {\mathcal{I}}_{\mathcal{A}}\left(\mathcal{x}\right), {\mathcal{F}}_{\mathcal{A}}\left(\mathcal{x}\right)\right) \rangle |\mathcal{x}\in \mathcal{S}, \left({\mathcal{T}}_{\mathcal{A}}\left(\mathcal{x}\right), {\mathcal{I}}_{\mathcal{A}}\left(\mathcal{x}\right), {\mathcal{F}}_{\mathcal{A}}\left(\mathcal{x}\right)\right)\in {]}^{-}\mathrm{0,1}{[}^{+})\}$$

2 $${0}^{-}\le {\mathcal{T}}_{\mathcal{A}}\left(\mathcal{x}\right)+{\mathcal{I}}_{\mathcal{A}}\left(\mathcal{x}\right)+{\mathcal{F}}_{\mathcal{A}}\left(\mathcal{x}\right)\le {3}^{+}$$

3 $$\mathcal{A}=\{\langle \mathcal{x}, \left({\mathcal{T}}_{\mathcal{A}}\left(\mathcal{x}\right), {\mathcal{I}}_{\mathcal{A}}\left(\mathcal{x}\right), {\mathcal{F}}_{\mathcal{A}}\left(\mathcal{x}\right)\right) \rangle |\mathcal{x}\in \mathcal{S}, \left({\mathcal{T}}_{\mathcal{A}}\left(\mathcal{x}\right), {\mathcal{I}}_{\mathcal{A}}\left(\mathcal{x}\right), {\mathcal{F}}_{\mathcal{A}}\left(\mathcal{x}\right)\right)\in \{\mathrm{0,1}])\}$$

#### Definition (Interval-valued neutrosophic set) [68]

 An interval-valued neutrosophic set (IVNS) is defined as follows:

4 $$\begin{aligned}{ \mathcal{A}} = \left\{ {\mathcal{x}, \left[ {{ \mathcal{T}}_{{ \mathcal{A}}}^{{ \mathcal{L}}} \left( \mathcal{x} \right),{ \mathcal{T}}_{{ \mathcal{A}}}^{{ \mathcal{U}}} \left( \mathcal{x} \right)} \right], [{ \mathcal{I}}_{{ \mathcal{A}}}^{{ \mathcal{L}}} \left( \mathcal{x} \right),{ \mathcal{I}}_{{ \mathcal{A}}}^{{ \mathcal{U}}} \left( \mathcal{x} \right), \left[ {{ \mathcal{F}}_{{ \mathcal{A}}}^{{ \mathcal{L}}} \left( \mathcal{x} \right),{ \mathcal{F}}_{{ \mathcal{A}}}^{{ \mathcal{U}}} \left( \mathcal{x} \right)} \right] |\mathcal{x} \in { \mathcal{S}}} \right\} \end{aligned}$$

Like those of single valued neutrosophic numbers, the values of the functions must be between 0 and 1.

The selection of an interval-valued neutrosophic (IVN) environment in this study is motivated by the characteristics of the green hydrogen dataset, which combines verified numerical indicators with forward-looking, partially realized information. Unlike probabilistic approaches that require large historical samples and distributional assumptions, IVN modeling does not depend on prior probability structures. Furthermore, classical fuzzy and intuitionistic fuzzy models are limited in handling situations where the degree of uncertainty cannot be fully resolved into membership and non-membership components. By introducing an explicit indeterminacy interval, IVN sets allow the proposed framework to accommodate data incompleteness, conflicting signals, and transitional uncertainty inherent in emerging energy technologies. Therefore, the IVN framework provides a theoretically consistent and practically suitable uncertainty representation for evaluating green hydrogen performance at the national level.

#### Definition (Operations of IVN Sets) [69–72]

Let $\widetilde{\mathfrak{m}}=\langle \left[{\mathcal{T}}_{\mathfrak{m}}^{\mathcal{L}},{\mathcal{T}}_{\mathfrak{m}}^{\mathcal{U}}\right], [{\mathcal{I}}_{\mathfrak{m}}^{\mathcal{L}},{\mathcal{I}}_{\mathfrak{m}}^{\mathcal{U}}], [{\mathcal{F}}_{\mathfrak{m}}^{\mathcal{L}},{\mathcal{F}}_{\mathfrak{m}}^{\mathcal{U}}]\rangle$ and $\widetilde{\mathfrak{n}}=\langle \left[{\mathcal{T}}_{\mathfrak{n}}^{\mathcal{L}},{\mathcal{T}}_{\mathfrak{n}}^{\mathcal{U}}\right], [{\mathcal{I}}_{\mathfrak{n}}^{\mathcal{L}},{\mathcal{I}}_{\mathfrak{n}}^{\mathcal{U}}], [{\mathcal{F}}_{\mathfrak{n}}^{\mathcal{L}},{\mathcal{F}}_{\mathfrak{n}}^{\mathcal{U}}]\rangle$ be two IVN numbers. Basic mathematical operations on $\widetilde{\mathfrak{m}}$ and $\widetilde{\mathfrak{n}}$ are given as follows.

(i) $$\widetilde{\mathfrak{m}}+\widetilde{\mathfrak{n}}=\langle \left[{\mathcal{T}}_{\mathfrak{m}}^{\mathcal{L}}+{\mathcal{T}}_{\mathfrak{n}}^{\mathcal{L}}-{\mathcal{T}}_{\mathfrak{m}}^{\mathcal{L}}{\mathcal{T}}_{\mathfrak{n}}^{\mathcal{L}}, {\mathcal{T}}_{\mathfrak{m}}^{\mathcal{U}}+{\mathcal{T}}_{\mathfrak{n}}^{\mathcal{U}}-{\mathcal{T}}_{\mathfrak{m}}^{\mathcal{U}}{\mathcal{T}}_{\mathfrak{n}}^{\mathcal{U}} \right], \left[{\mathcal{I}}_{\mathfrak{m}}^{\mathcal{L}}{\mathcal{I}}_{\mathfrak{n}}^{\mathcal{L}}, {\mathcal{I}}_{\mathfrak{m}}^{\mathcal{U}}{\mathcal{I}}_{\mathfrak{n}}^{\mathcal{U}}\right], \left[{\mathcal{F}}_{\mathfrak{m}}^{\mathcal{L}}{\mathcal{F}}_{\mathfrak{n}}^{\mathcal{L}}, {\mathcal{F}}_{\mathfrak{m}}^{\mathcal{U}}{\mathcal{F}}_{\mathfrak{n}}^{\mathcal{U}}\right]\rangle $$
(ii) $$\widetilde{\mathfrak{m}}-\widetilde{\mathfrak{n}}=\langle \left[{\mathcal{T}}_{\mathfrak{m}}^{\mathcal{L}}-{\mathcal{F}}_{\mathfrak{n}}^{\mathcal{U}}, {\mathcal{T}}_{\mathfrak{m}}^{\mathcal{U}}-{\mathcal{F}}_{\mathfrak{n}}^{\mathcal{L}}\right], \left[max\left({\mathcal{T}}_{\mathfrak{m}}^{\mathcal{L}}{\mathcal{T}}_{\mathfrak{n}}^{\mathcal{L}}\right),max({\mathcal{T}}_{\mathfrak{m}}^{\mathcal{U}}{\mathcal{T}}_{\mathfrak{n}}^{\mathcal{U}})\right], \left[{\mathcal{F}}_{\mathfrak{m}}^{\mathcal{L}}-{\mathcal{T}}_{\mathfrak{n}}^{\mathcal{U}}, {\mathcal{F}}_{\mathfrak{m}}^{\mathcal{U}}-{\mathcal{T}}_{\mathfrak{n}}^{\mathcal{L}}\right]\rangle $$
(iii) $$\widetilde{\mathfrak{m}}\times \widetilde{\mathfrak{n}}=\langle \left[{\mathcal{T}}_{\mathfrak{m}}^{\mathcal{L}}{\mathcal{T}}_{\mathfrak{n}}^{\mathcal{L}}, {\mathcal{T}}_{\mathfrak{m}}^{\mathcal{U}}{\mathcal{T}}_{\mathfrak{n}}^{\mathcal{U}}\right], \left[{\mathcal{I}}_{\mathfrak{m}}^{\mathcal{L}}+{\mathcal{I}}_{\mathfrak{n}}^{\mathcal{L}}-{\mathcal{I}}_{\mathfrak{m}}^{\mathcal{L}}{\mathcal{I}}_{\mathfrak{n}}^{\mathcal{L}}, {\mathcal{I}}_{\mathfrak{m}}^{\mathcal{U}}+{\mathcal{I}}_{\mathfrak{n}}^{\mathcal{U}}-{\mathcal{I}}_{\mathfrak{m}}^{\mathcal{U}}{\mathcal{I}}_{\mathfrak{n}}^{\mathcal{U}}\right], $$$$\left[{\mathcal{F}}_{\mathfrak{m}}^{\mathcal{L}}+{\mathcal{F}}_{\mathfrak{n}}^{\mathcal{L}}-{\mathcal{F}}_{\mathfrak{m}}^{\mathcal{L}}{\mathcal{F}}_{\mathfrak{n}}^{\mathcal{L}}, {\mathcal{F}}_{\mathfrak{m}}^{\mathcal{U}}+{\mathcal{F}}_{\mathfrak{n}}^{\mathcal{U}}-{\mathcal{F}}_{\mathfrak{m}}^{\mathcal{U}}{\mathcal{F}}_{\mathfrak{n}}^{\mathcal{U}}\right]\rangle$$   
(iv) $${\left(\widetilde{\mathfrak{m}}\right)}^{\lambda }=\langle \left[{\left({\mathcal{T}}_{\mathfrak{m}}^{\mathcal{L}}\right)}^{\lambda }, {\left({\mathcal{T}}_{\mathfrak{m}}^{\mathcal{U}}\right)}^{\lambda }\right],\left[{\left({\mathcal{I}}_{\mathfrak{m}}^{\mathcal{L}}\right)}^{\lambda }, {\left({\mathcal{I}}_{\mathfrak{m}}^{\mathcal{U}}\right)}^{\lambda }\right], \left[{1-\left(1-{\mathcal{F}}_{\mathfrak{m}}^{\mathcal{L}}\right)}^{\lambda }, {1-\left(1-{\mathcal{F}}_{\mathfrak{m}}^{\mathcal{U}}\right)}^{\lambda }\right]\rangle ; \lambda \ge 0$$
(v) $$\lambda \widetilde{\mathfrak{m}}=\langle \left[{1-\left(1-{\mathcal{T}}_{\mathfrak{m}}^{\mathcal{L}}\right)}^{\lambda }, {1-\left(1-{\mathcal{T}}_{\mathfrak{m}}^{\mathcal{U}}\right)}^{\lambda }\right],\left[{\left({\mathcal{I}}_{\mathfrak{m}}^{\mathcal{L}}\right)}^{\lambda }, {\left({\mathcal{I}}_{\mathfrak{m}}^{\mathcal{U}}\right)}^{\lambda }\right], \left[{\left({\mathcal{F}}_{\mathfrak{m}}^{\mathcal{L}}\right)}^{\lambda }, {\left({\mathcal{F}}_{\mathfrak{m}}^{\mathcal{U}}\right)}^{\lambda }\right]\rangle ; \lambda \ge 0$$
(vi) $\widetilde{\mathfrak{m}}\subseteq \widetilde{\mathfrak{n}}$ if and only if ${\mathcal{T}}_{\mathfrak{m}}^{\mathcal{L}}\le {\mathcal{T}}_{\mathfrak{n}}^{\mathcal{L}}, {\mathcal{T}}_{\mathfrak{m}}^{\mathcal{U}}\le {\mathcal{T}}_{\mathfrak{n}}^{\mathcal{U}}$;${\mathcal{I}}_{\mathfrak{m}}^{\mathcal{L}}\ge {\mathcal{I}}_{\mathfrak{n}}^{\mathcal{L}}, {\mathcal{I}}_{\mathfrak{m}}^{\mathcal{U}}\ge {\mathcal{I}}_{\mathfrak{n}}^{\mathcal{U}}; {\mathcal{F}}_{\mathfrak{m}}^{\mathcal{L}}\ge {\mathcal{F}}_{\mathfrak{n}}^{\mathcal{L}}, {\mathcal{F}}_{\mathfrak{m}}^{\mathcal{U}}\ge {\mathcal{F}}_{\mathfrak{n}}^{\mathcal{U}}$
(vii) ${\left(\widetilde{\mathfrak{m}}\right)}^{\mathcal{c}}=\langle \left[{\mathcal{F}}_{\mathfrak{m}}^{\mathcal{L}},{\mathcal{F}}_{\mathfrak{m}}^{\mathcal{U}}\right],\left[1-{\mathcal{I}}_{\mathfrak{m}}^{\mathcal{L}},1-{\mathcal{I}}_{\mathfrak{m}}^{\mathcal{U}}\right], \left[{\mathcal{T}}_{\mathfrak{m}}^{\mathcal{L}},{\mathcal{T}}_{\mathfrak{m}}^{\mathcal{U}}\right]\rangle , {\left(\widetilde{\mathfrak{m}}\right)}^{\mathcal{c}}$ is called the complement of $\widetilde{\mathfrak{m}}.$

#### Definition (Measure of entropy value) [47]

5 $$\text{A function }\mathcal{E}:IVNS(\mathcal{x})\to \left[\mathrm{0,1}\right]\text{ with }\mathcal{E}\left(\mathcal{A}\right)=\frac{1-{\mathcal{u}}_{\mathcal{A}}}{1+{\mathcal{u}}_{\mathcal{A}}}$$

where   

$${\mathcal{u}}_{\mathcal{A}}=\frac{1}{4\mathcal{n}}\sum_{\mathcal{i}=1}^{\mathcal{n}}\left[\begin{array}{c}\left|{\mathcal{T}}_{\mathcal{A}}^{\mathcal{L}}\left({\mathcal{x}}_{\mathcal{i}}\right)-0.5\right|+\left|{\mathcal{T}}_{\mathcal{A}}^{\mathcal{U}}\left({\mathcal{x}}_{\mathcal{i}}\right)-0.5\right|+\left|{\mathcal{I}}_{\mathcal{A}}^{\mathcal{L}}\left({\mathcal{x}}_{\mathcal{i}}\right)-0.5\right|+\left|{\mathcal{I}}_{\mathcal{A}}^{\mathcal{U}}\left({\mathcal{x}}_{\mathcal{i}}\right)-0.5\right|+\\ \left|{\mathcal{I}}_{{\mathcal{A}}^{\mathcal{c}}}^{\mathcal{L}}\left({\mathcal{x}}_{\mathcal{i}}\right)-0.5\right|+\left|{\mathcal{I}}_{{\mathcal{A}}^{\mathcal{c}}}^{\mathcal{U}}\left({\mathcal{x}}_{\mathcal{i}}\right)-0.5\right|+\left|{\mathcal{F}}_{\mathcal{A}}^{\mathcal{L}}\left({\mathcal{x}}_{\mathcal{i}}\right)-0.5\right|+\left|{\mathcal{F}}_{\mathcal{A}}^{\mathcal{U}}\left({\mathcal{x}}_{\mathcal{i}}\right)-0.5\right|\end{array}\right]$$

is an entropy value on $IVNS\left(\mathcal{x}\right).$

### Proposed distance formula: normalized centroid–spread interval-valued neutrosophic distance measure (NC-SIVNDM)

In the context of MCDM with IVN sets, we define an NC-SIVNDM between two alternatives A and B as follows:

For each criterion k and each neutrosophic component $\mathrm{M}\in \{\mathrm{T},\text{ I},\text{ F}\}$,

Let ${{\mathrm{M}}_{\mathrm{X}}}^{\left(\mathrm{k}\right)}=\left[{\mathrm{M}}_{\mathrm{X}}^{\mathrm{L}(\mathrm{k})},{\mathrm{M}}_{\mathrm{X}}^{\mathrm{U}(\mathrm{k})}\right]$ be the interval-valued membership for alternative $\mathrm{X}$, where ${\mathrm{M}}_{\mathrm{X}}^{\mathrm{L}(\mathrm{k})}$ is the lower bound and ${\mathrm{M}}_{\mathrm{X}}^{\mathrm{U}(\mathrm{k})}$ is the upper bound.

We define the centroid and spread as

- Centroid: $${{\mathrm{C}}_{\mathrm{M}}}^{\left(\mathrm{k}\right)}\left(\mathrm{X}\right)=\frac{{\mathrm{M}}_{\mathrm{X}}^{\mathrm{L}(\mathrm{k})}+{\mathrm{M}}_{\mathrm{X}}^{\mathrm{U}(\mathrm{k})}}{2},$$
- Spread: $${{\mathrm{S}}_{\mathrm{M}}}^{\left(\mathrm{k}\right)}\left(\mathrm{X}\right)=\frac{{\mathrm{M}}_{\mathrm{X}}^{\mathrm{U}(\mathrm{k})}-{\mathrm{M}}_{\mathrm{X}}^{\mathrm{L}(\mathrm{k})}}{2}$$
  6 $${\mathrm{D}}_{\mathrm{NCSIVN}}=\frac{2}{\sqrt{15\mathrm{n}}}\sqrt{\sum_{\mathrm{k}=1}^{\mathrm{n}}{\sum }_{\mathrm{M}\in \left\{\mathrm{T},\mathrm{I},\mathrm{F}\right\}}{({({\mathrm{C}}_{\mathrm{M}}}^{\left(\mathrm{k}\right)}\left(\mathrm{X}\right)-{({\mathrm{C}}_{\mathrm{M}}}^{\left(\mathrm{k}\right)}\left(\mathrm{Y}\right))}^{2}+{{({\mathrm{S}}_{\mathrm{M}}}^{\left(\mathrm{k}\right)}\left(\mathrm{X}\right)-{({\mathrm{S}}_{\mathrm{M}}}^{\left(\mathrm{k}\right)}\left(\mathrm{Y}\right))}^{2}})$$

For each neutrosophic set X, Y ∈ {A, B, C}, define the centroid-spread Vectors ${\mathrm{P}}_{\mathrm{X}}$ and ${\mathrm{P}}_{\mathrm{Y}}$:

$$\begin{aligned}{\mathrm{P}}_{\mathrm{X}}=&\;({{\mathrm{C}}_{\mathrm{T}}}^{1}\left(\mathrm{X}\right),{{\mathrm{C}}_{\mathrm{I}}}^{1}\left(\mathrm{X}\right),{{\mathrm{C}}_{\mathrm{F}}}^{1}\left(\mathrm{X}\right),{{\mathrm{S}}_{\mathrm{T}}}^{1}\left(\mathrm{X}\right),{{\mathrm{S}}_{\mathrm{I}}}^{1}\left(\mathrm{X}\right),{{\mathrm{S}}_{\mathrm{F}}}^{1}\left(\mathrm{X}\right),\dots ,\\&\quad{{\mathrm{C}}_{\mathrm{T}}}^{\mathrm{n}}\left(\mathrm{X}\right),{{\mathrm{C}}_{\mathrm{I}}}^{\mathrm{n}}\left(\mathrm{X}\right),{{\mathrm{C}}_{\mathrm{F}}}^{\mathrm{n}}\left(\mathrm{X}\right),{{\mathrm{S}}_{\mathrm{T}}}^{\mathrm{n}}\left(\mathrm{X}\right),{{\mathrm{S}}_{\mathrm{I}}}^{\mathrm{n}}\left(\mathrm{X}\right),{{\mathrm{S}}_{\mathrm{F}}}^{\mathrm{n}}\left(\mathrm{X}\right))\in {\mathbb{R}}^{6\mathrm{n}}\end{aligned}$$

and

$$\begin{aligned}{\mathrm{P}}_{\mathrm{Y}}=&\;({{\mathrm{C}}_{\mathrm{T}}}^{1}\left(\mathrm{Y}\right),{{\mathrm{C}}_{\mathrm{I}}}^{1}\left(\mathrm{Y}\right),{{\mathrm{C}}_{\mathrm{F}}}^{1}\left(\mathrm{Y}\right),{{\mathrm{S}}_{\mathrm{T}}}^{1}\left(\mathrm{Y}\right),{{\mathrm{S}}_{\mathrm{I}}}^{1}\left(\mathrm{Y}\right),{{\mathrm{S}}_{\mathrm{F}}}^{1}\left(\mathrm{Y}\right),\dots ,\\& \quad{{\mathrm{C}}_{\mathrm{T}}}^{\mathrm{n}}\left(\mathrm{Y}\right),{{\mathrm{C}}_{\mathrm{I}}}^{\mathrm{n}}\left(\mathrm{Y}\right),{{\mathrm{C}}_{\mathrm{F}}}^{\mathrm{n}}\left(\mathrm{Y}\right),{{\mathrm{S}}_{\mathrm{T}}}^{\mathrm{n}}\left(\mathrm{Y}\right),{{\mathrm{S}}_{\mathrm{I}}}^{\mathrm{n}}\left(\mathrm{Y}\right),{{\mathrm{S}}_{\mathrm{F}}}^{\mathrm{n}}\left(\mathrm{Y}\right))\in {\mathbb{R}}^{6\mathrm{n}}\end{aligned}$$

Here, the interval components are flattened into vector entries to allow direct Euclidean computation. These flattening preserves component wise relationships because each triplet corresponds to an independent dimension in the vector space.

1. Non negativity

Clearly,   

$$\sqrt{\sum_{\mathrm{k}=1}^{\mathrm{n}}{\sum }_{\mathrm{M}\in \left\{\mathrm{T},\mathrm{I},\mathrm{F}\right\}}{({({\mathrm{C}}_{\mathrm{M}}}^{\left(\mathrm{k}\right)}\left(\mathrm{X}\right)-{({\mathrm{C}}_{\mathrm{M}}}^{\left(\mathrm{k}\right)}\left(\mathrm{Y}\right))}^{2}+{{({\mathrm{S}}_{\mathrm{M}}}^{\left(\mathrm{k}\right)}\left(\mathrm{X}\right)-{({\mathrm{S}}_{\mathrm{M}}}^{\left(\mathrm{k}\right)}\left(\mathrm{Y}\right))}^{2}})\ge 0$$

for all vectors in ${\mathrm{R}}^{6\mathrm{n}}$ and $\frac{2}{\sqrt{15n}}\ge 0$

$$\begin{aligned}&\Rightarrow {\mathrm{D}}_{\mathrm{NCSIVN}}\left(\mathrm{X},\mathrm{Y}\right)=\frac{2}{\sqrt{15n}}\sqrt{\sum_{\mathrm{k}=1}^{\mathrm{n}}{\sum }_{\mathrm{M}\in \left\{\mathrm{T},\mathrm{I},\mathrm{F}\right\}}{({({\mathrm{C}}_{\mathrm{M}}}^{\left(\mathrm{k}\right)}\left(\mathrm{A}\right)-{({\mathrm{C}}_{\mathrm{M}}}^{\left(\mathrm{k}\right)}\left(\mathrm{B}\right))}^{2}+{{({\mathrm{S}}_{\mathrm{M}}}^{\left(\mathrm{k}\right)}\left(\mathrm{A}\right)-{({\mathrm{S}}_{\mathrm{M}}}^{\left(\mathrm{k}\right)}\left(\mathrm{B}\right))}^{2}})\ge 0\\&\;\Rightarrow{\mathrm{D}}_{\mathrm{NCSIVN}}(\mathrm{X},\mathrm{Y})\ge 0\end{aligned}$$

(2) Identity of indiscernibility

${\mathrm{D}}_{\mathrm{NCSIVN}}\left(\mathrm{X},\mathrm{Y}\right)=0$, if and only if

$${{\mathrm{C}}^{(\mathrm{k})}}_{\mathrm{M}}\left(\mathrm{X}\right)$$= $${{\mathrm{C}}^{(\mathrm{k})}}_{\mathrm{M}}\left(\mathrm{Y}\right)$$ and $${{\mathrm{S}}^{(\mathrm{k})}}_{\mathrm{M}}\left(\mathrm{X}\right)$$ = $${{\mathrm{S}}^{(\mathrm{k})}}_{\mathrm{M}}\left(\mathrm{Y}\right)$$ $$\forall \text{ k}=\mathrm{1,2},\dots ,\mathrm{n},\text{ M}\in \left\{\mathrm{T},\mathrm{I},\mathrm{F}\right\}$$

${\mathrm{D}}_{\mathrm{NCSIVN}}\left(\mathrm{X},\mathrm{Y}\right)=0$ ⟺$\mathrm{X}=\mathrm{Y}$ in terms of their neutrosophic components.

(3) Symmetry

For any $$\mathrm{X},\mathrm{Y}:$$

$$\begin{aligned}{\mathrm{D}}_{\mathrm{NCSIVN}}\left(\mathrm{X},\mathrm{Y}\right)=&\;\frac{2}{\sqrt{15\mathrm{n}}}\sqrt{\sum_{\mathrm{k}=1}^{\mathrm{n}}{\sum }_{\mathrm{M}\in \left\{\mathrm{T},\mathrm{I},\mathrm{F}\right\}}{({({\mathrm{C}}_{\mathrm{M}}}^{\left(\mathrm{k}\right)}\left(\mathrm{X}\right)-{({\mathrm{C}}_{\mathrm{M}}}^{\left(\mathrm{k}\right)}\left(\mathrm{Y}\right))}^{2}+{{({\mathrm{S}}_{\mathrm{M}}}^{\left(\mathrm{k}\right)}\left(\mathrm{X}\right)-{({\mathrm{S}}_{\mathrm{M}}}^{\left(\mathrm{k}\right)}\left(\mathrm{Y}\right))}^{2}})\\=&\;\frac{2}{\sqrt{15\mathrm{n}}}\sqrt{\sum_{\mathrm{k}=1}^{\mathrm{n}}{\sum }_{\mathrm{M}\in \left\{\mathrm{T},\mathrm{I},\mathrm{F}\right\}}{({({\mathrm{C}}_{\mathrm{M}}}^{\left(\mathrm{k}\right)}\left(\mathrm{Y}\right)-{({\mathrm{C}}_{\mathrm{M}}}^{\left(\mathrm{k}\right)}\left(\mathrm{X}\right))}^{2}+{{({\mathrm{S}}_{\mathrm{M}}}^{\left(\mathrm{k}\right)}\left(\mathrm{Y}\right)-{({\mathrm{S}}_{\mathrm{M}}}^{\left(\mathrm{k}\right)}\left(\mathrm{X}\right))}^{2}}\\=&\;{\mathrm{D}}_{\mathrm{NCSIVN}}\left(\mathrm{Y},\mathrm{X}\right)\end{aligned}$$

(4) Triangle inequality

Then, the squared Euclidean distance between any two such vectors is

$$\mathrm{S}\left(\mathrm{X},\mathrm{Y}\right)={\sum_{\mathrm{k}=1}^{\mathrm{n}}\sum_{\mathrm{M}\in \left\{\mathrm{T},\mathrm{I},\mathrm{F}\right\}}[({{\mathrm{C}}^{(\mathrm{k})}}_{\mathrm{M}}\left(\mathrm{X}\right)-{{\mathrm{C}}^{(\mathrm{k})}}_{\mathrm{M}}\left(\mathrm{Y}\right))}^{2}+{{({\mathrm{S}}^{(\mathrm{k})}}_{\mathrm{M}}\left(\mathrm{X}\right)-{({\mathrm{S}}^{\mathrm{k})}}_{\mathrm{M}}\left(\mathrm{Y}\right))}^{2}]$$

Note that the Euclidean norm in ${\mathbb{R}}^{6\mathrm{n}}$ satisfies it, but we derive this explicitly for completeness.

So $${\mathrm{D}}_{\mathrm{NCSIVN}}\left(\mathrm{X},\mathrm{Y}\right)=\frac{2}{\sqrt{15\mathrm{n}}}\sqrt{\mathrm{S}\left(\mathrm{X},\mathrm{Y}\right)}$$

⇒To confirm that ${\mathrm{D}}_{\mathrm{NCSIVN}}$ is a metric, it suffices to show.

$$\sqrt{\mathrm{S}\left(\mathrm{X},\mathrm{Z}\right)}\le \sqrt{\mathrm{S}\left(\mathrm{X},\mathrm{Y}\right)}+\sqrt{\mathrm{S}\left(\mathrm{Y},\mathrm{Z}\right)}.$$

Each component ${\mathrm{z}}_{\mathrm{k},\mathrm{M}}$ represents one entry in the vector ${\mathrm{P}}_{\mathrm{X}}$ (e.g., a specific ${{\mathrm{C}}_{\mathrm{T}}}^{\left(\mathrm{k}\right)}\left(\mathrm{X}\right)\text{ or }{{\mathrm{S}}_{\mathrm{T}}}^{\left(\mathrm{k}\right)}\left(\mathrm{X}\right))$

For simplicity, we flatten all $6\mathrm{n}$ of them into indices $\mathrm{i}=1$ to $6\mathrm{n}$, but keeping $\mathrm{k}$ and $\mathrm{M}$ makes it match your formula.

Let $${\mathrm{a}}_{\mathrm{k},\mathrm{M}}={\mathrm{Z}}_{\mathrm{k},\mathrm{M}}\left(\mathrm{X}\right)-{\mathrm{Z}}_{\mathrm{k},\mathrm{M}}\left(\mathrm{Y}\right)$$ and $${\mathrm{b}}_{\mathrm{k},\mathrm{M}}={\mathrm{Z}}_{\mathrm{k},\mathrm{M}}\left(\mathrm{Y}\right)-{\mathrm{Z}}_{\mathrm{k},\mathrm{M}}\left(\mathrm{Z}\right)$$

$$\Rightarrow {\mathrm{Z}}_{\mathrm{k},\mathrm{M}}\left(\mathrm{X}\right)-{\mathrm{Z}}_{\mathrm{k},\mathrm{M}}\left(\mathrm{Z}\right){[\mathrm{Z}}_{\mathrm{k},\mathrm{M}}\left(\mathrm{X}\right)-{\mathrm{Z}}_{\mathrm{k},\mathrm{M}}\left(\mathrm{Y}\right)]+{[\mathrm{Z}}_{\mathrm{k},\mathrm{M}}\left(\mathrm{Y}\right)-{\mathrm{Z}}_{\mathrm{k},\mathrm{M}}\left(\mathrm{Z}\right)]= {\mathrm{a}}_{\mathrm{k},\mathrm{M}}+{\mathrm{b}}_{\mathrm{k},\mathrm{M}}$$

$$\Rightarrow \mathrm{S}\left(\mathrm{X},\mathrm{Z}\right)=\sum_{\mathrm{k}=1}^{\mathrm{n}}\sum_{\mathrm{M}\in \left\{\mathrm{T},\mathrm{I},\mathrm{F}\right\}}{{[\mathrm{Z}}_{\mathrm{k},\mathrm{M}}\left(\mathrm{X}\right)-{\mathrm{Z}}_{\mathrm{k},\mathrm{M}}\left(\mathrm{Z}\right)]}^{2}=\sum_{\mathrm{k}=1}^{\mathrm{n}}\sum_{\mathrm{M}\in \left\{\mathrm{T},\mathrm{I},\mathrm{F}\right\}}{({\mathrm{a}}_{\mathrm{k},\mathrm{M}}+{\mathrm{b}}_{\mathrm{k},\mathrm{M}})}^{2}$$

$$=\sum_{\mathrm{k}=1}^{\mathrm{n}}\sum_{\mathrm{M}\in \left\{\mathrm{T},\mathrm{I},\mathrm{F}\right\}}{{\mathrm{a}}^{2}}_{\mathrm{k},\mathrm{M}}+{{\mathrm{b}}^{2}}_{\mathrm{k},\mathrm{M}}+2{(\mathrm{a}}_{\mathrm{k},\mathrm{M}})({\mathrm{b}}_{\mathrm{k},\mathrm{M}})$$

7 $$\Rightarrow \mathrm{S}\left(\mathrm{X},\mathrm{Z}\right)=\mathrm{S}\left(\mathrm{X},\mathrm{Y}\right)+\mathrm{S}\left(\mathrm{Y},\mathrm{Z}\right)+2\sum_{\mathrm{k}=1}^{\mathrm{n}}\sum_{\mathrm{M}\in \left\{\mathrm{T},\mathrm{I},\mathrm{F}\right\}}{(\mathrm{a}}_{\mathrm{k},\mathrm{M}})({\mathrm{b}}_{\mathrm{k},\mathrm{M}})$$

By the Cauchy–Schwarz inequality, for any two real numbers $\mathrm{p},\mathrm{q}$

$$\left|\sum \mathrm{pq}\right|\le \sqrt{\sum {\mathrm{p}}^{2}}.\sqrt{\sum {\mathrm{q}}^{2}}.$$

Flattening the double sum to a single sum over $6\mathrm{n}$ terms:

$$\left|\sum_{\mathrm{k}=1}^{\mathrm{n}}\sum_{\mathrm{M}\in \left\{\mathrm{T},\mathrm{I},\mathrm{F}\right\}}{(\mathrm{a}}_{\mathrm{k},\mathrm{M}})({\mathrm{b}}_{\mathrm{k},\mathrm{M}})\right|\le \sqrt{\sum_{\mathrm{k}=1}^{\mathrm{n}}\sum_{\mathrm{M}\in \left\{\mathrm{T},\mathrm{I},\mathrm{F}\right\}}{{\mathrm{a}}^{2}}_{\mathrm{k},\mathrm{M}}}.\sqrt{\sum_{\mathrm{k}=1}^{\mathrm{n}}\sum_{\mathrm{M}\in \left\{\mathrm{T},\mathrm{I},\mathrm{F}\right\}}{{\mathrm{b}}^{2}}_{\mathrm{k},\mathrm{M}}}$$

8 $$ \mathop \sum \limits_{{{\mathrm{k}} = 1}}^{{\mathrm{n}}} \mathop \sum \limits_{{{\mathrm{M}} \in \left\{ {{\mathrm{T}},{\mathrm{I}},{\mathrm{F}}} \right\}}} ({\mathrm{a}}_{{{\mathrm{k}},{\mathrm{M}}}} )\left( {{\mathrm{b}}_{{{\mathrm{k}},{\mathrm{M}}}} } \right) \le \sqrt {{\mathrm{S}}\left( {{\mathrm{X}},{\mathrm{Y}}} \right) \cdot {\mathrm{S}}\left( {{\mathrm{Y}},{\mathrm{Z}}} \right)} $$

From (7) and (8), We have

$$\begin{aligned}\mathrm{S}\left(\mathrm{X},\mathrm{Z}\right)\le&\; \mathrm{S}\left(\mathrm{X},\mathrm{Y}\right)+\mathrm{S}\left(\mathrm{Y},\mathrm{Z}\right)+2\sqrt{\mathrm{S}\left(\mathrm{X},\mathrm{Y}\right).\mathrm{S}\left(\mathrm{Y},\mathrm{Z}\right)}\\=&\;{\sqrt{\mathrm{S}\left(\mathrm{X},\mathrm{Y}\right)}}^{2}+{\sqrt{\mathrm{S}\left(\mathrm{Y},\mathrm{Z}\right)}}^{2}+2\sqrt{\mathrm{S}\left(\mathrm{X},\mathrm{Y}\right)} .\sqrt{\mathrm{S}\left(\mathrm{Y},\mathrm{Z}\right)}\\=&\;{\left(\sqrt{\mathrm{S}\left(\mathrm{X},\mathrm{Y}\right)}+\sqrt{\mathrm{S}\left(\mathrm{Y},\mathrm{Z}\right)}\right)}^{2}\\\mathrm{S}\left(\mathrm{X},\mathrm{Z}\right)\le&\; {\left(\sqrt{\mathrm{S}\left(\mathrm{X},\mathrm{Y}\right)}+\sqrt{\mathrm{S}\left(\mathrm{Y},\mathrm{Z}\right)}\right)}^{2}\end{aligned}$$

Take the square root on both sides,

$$\sqrt{\mathrm{S}\left(\mathrm{X},\mathrm{Z}\right)}\le \sqrt{\mathrm{S}\left(\mathrm{X},\mathrm{Y}\right)}+\sqrt{\mathrm{S}\left(\mathrm{Y},\mathrm{Z}\right)}$$

Both sides are multiplied by $\frac{2}{\sqrt{15\mathrm{n}}}$

$$\frac{2}{\sqrt{15\mathrm{n}}}\sqrt{\mathrm{S}\left(\mathrm{X},\mathrm{Z}\right)}\le \frac{2}{\sqrt{15\mathrm{n}}}\sqrt{\mathrm{S}\left(\mathrm{X},\mathrm{Y}\right)}+\frac{2}{\sqrt{15\mathrm{n}}}\sqrt{\mathrm{S}\left(\mathrm{Y},\mathrm{Z}\right)}$$

$$\Rightarrow{\mathrm{D}}_{\mathrm{NCSIVN}}(\mathrm{X},\mathrm{Z})\le {\mathrm{D}}_{\mathrm{NCSIVN}}(\mathrm{X},\mathrm{Y})+{\mathrm{D}}_{\mathrm{NCSIVN}}(\mathrm{Y},\mathrm{Z}).$$

Thus, the proposed distance measure $\mathrm{D}$ satisfies the triangle inequality.

### IVN-entropy objective weighting method:

Entropy theory was first developed by Shannon to quantify ambiguous information and entropy. The fundamental principle of entropy weight measurement is that a higher weight index value commands greater significance than a lower index value. This method was implemented to ascertain the objective weights allocated to every criterion, reflecting their significance and considering the variability of the preliminary information [73, 74]. The subsequent stages outline the application of the entropy objective weighting method to MCDM [56].

*Step 1.* The preliminary interval-valued neutrosophic decision matrix ($\widetilde{\mathcal{X}}$) is constructed.

The quantitative results for each alternative ${\mathcal{A}}_{\mathcal{i}}$ for every criterion ${\mathcal{C}}_{\mathcal{j}}$ are gathered from trustworthy secondary sources and transformed into interval-valued neutrosophic numbers (IVNNs) utilizing an established linguistic or numerical mapping scale, as illustrated in Eq. (9).9

Here, ${\stackrel{`}{\mathcal{x}}}_{\mathcal{i}\mathcal{j}}$ represents the assessment score of the ${\mathcal{i}}^{th}$ alternative relative to the ${\mathcal{j}}^{th}$ criterion, where $\mathcal{m}$ denotes the total number of alternatives and where $\mathcal{n}$ indicates the total number of criteria.

Table 2 illustrates the conversion of linguistic terms, including Very Low, Low, Moderate, High, and Very High, into IVNNs. Each term is distinguished by intervals of truth ($\mathcal{T}$), indeterminacy ($\mathcal{I}$), and falsity ($\mathcal{F}$) values within the range of [0,1], enabling qualitative interpretations to be quantitatively expressed in the IVN framework.

**Table 2:** Correlation between linguistic variables and the IVN scale [47]

Linguistic variables	Interval neutrosophic numbers
Very low (VL)	([0.1,0.2], [0.7,0.8], [0.6,0.7])
Low (L)	([0.3,0.4], [0.6,0.7], [0.5,0.6])
Medium (M)	([0.4,0.5], [0.5,0.6], [0.4,0.5])
High (H)	([0.6,0.7], [0.4,0.5], [0.3,0.4])
Very high (VH)	([0.7,0.8], [0.2,0.3], [0.1,0.2])

*Step 2:* To obtain the weights of the criteria, we calculate the entropy of the criterion ${\mathcal{C}}_{\mathcal{j}}$ as defined in Eq. 5. The entropy ${\mathcal{E}}_{\mathcal{j}}$ represents the information content of the ${\mathcal{j}}^{th}$ criterion for all $\mathcal{j}=\mathrm{1,2},3,\dots , \mathcal{n}.$

*Step 3.* To determine the objective weighting (${\mathcal{W}}_{\mathcal{j}})$ for each criterion ${\mathcal{C}}_{\mathcal{j}}$, Eq. (10) is employed.

10 $${ \mathcal{W}}_{\mathcal{j}} = \frac{{1 - { \mathcal{E}}_{\mathcal{j}} }}{{\mathop \sum \nolimits_{\mathcal{j} = 1}^{\mathcal{j}} \left( {1 - { \mathcal{E}}_{\mathcal{j}} } \right)}} $$

The acquired objective weights are employed in the MARCOS model in the subsequent step to assess the performance of each alternative.

### Proposed NC-SIVNDM MARCOS ranking method

The MARCOS (measurement of alternatives and ranking according to compromise solution) method, originated by Stević et al. [49], is an MCDM framework that aims at evaluating and distributing alternatives by correlating them with reference values, notably the ideal and anti-ideal solutions. The method identifies the utility functions of alternatives on the basis of these relationships, which demonstrate the relative closeness of each alternative to the ideal and its corresponding distance from the anti-ideal reference point. The option exhibiting an outstanding utility function is deemed the most acceptable of preferences. MARCOS describes a systematic framework that integrates reference points, inter-alternative connections, and utility levels to facilitate rational and equitable decision-making in scenarios with various criteria and alternatives [75, 76]. The mathematical structure of the MARCOS approach is delineated as follows [57].

*Step 1* A preliminary decision-making matrix is developed using Eq. (11) that involves a set of $\mathcal{n}$ criteria and $\mathcal{m}$ alternatives [77].11where ${\stackrel{`}{\mathcal{x}}}_{\mathcal{i}\mathcal{j}}$ represents the performance of the ${\mathcal{i}}^{th}$ alternative concerning the ${\mathcal{j}}^{th}$ criterion, $\mathcal{m}$ symbolizes the number of alternatives, and $\mathcal{n}$ symbolizes the number of criteria.

*Step 2* An extended initial decision-making matrix is developed by outlining the ideal (AI) and anti-ideal (AAI) solutions via Eq. (12).12

The most beneficial (AI) solution demonstrates the option with the greatest efficiency, whereas the anti-optimal (AAI) solution represents the least favorable alternative. The definitions of the sets (AI) and (AAI) are contingent upon the nature of the criteria, as delineated in Eqs. (13) and (14).

13 $${ \mathbb{A}{\mathbb{A}}{\mathbb{I}}} = \min_{\mathcal{i}} \mathcal{x}_{\mathcal{ij}} , if \mathcal{j} \in { \mathcal{B}}\; and \max_{\mathcal{i}} \mathcal{x}_{\mathcal{ij}} , if \mathcal{j} \in { \mathcal{C}} $$

14 $${ \mathbb{A}{\mathbb{I}}} = \max_{\mathcal{i}} \mathcal{x}_{\mathcal{ij}} , if \mathcal{j} \in { \mathcal{B}}\; and \min_{\mathcal{i}} \mathcal{x}_{\mathcal{ij}} , if \mathcal{j} \in { \mathcal{C}} $$

where $\mathcal{B}$ represents a set of beneficial criteria and where $\mathcal{C}$ represents a set of non-beneficial criteria.

*Step 3* The extended initial matrix can be standardized using Eqs. (15) and (16), resulting in the normalized matrix $\mathcal{N}={\left[{\mathcal{n}}_{\mathcal{i}\mathcal{j}}\right]}_{\mathcal{m}\times \mathcal{n}}$.

15 $$\mathcal{n}_{\mathcal{ij}} = \frac{{\mathcal{x}_{\mathcal{ij}} }}{{\mathcal{x}_{\mathcal{ai}} }}, if \mathcal{j} \in { \mathcal{B}} $$

16 $${\mathcal{n}}_{\mathcal{i}\mathcal{j}}=\frac{{x}_{\mathcal{a}\mathcal{i}}}{{\mathrm{x}}_{\mathcal{i}\mathcal{j}}}, if \mathcal{j} \in \mathcal{C}$$

where the elements $\acute{\mathcal{x}}_{\mathcal{ij}}$ and $\acute{\mathcal{x}}_{\mathcal{ai}}$ denote the elements of the matrix $\widetilde{\mathcal{X}}$.

*Step 4* Determine the weighted matrix $\mathcal{V}={\left[{\mathcal{v}}_{\mathcal{i}\mathcal{j}}\right]}_{\mathcal{m}\times \mathcal{n}}$. The weighted matrix $\mathcal{V}$ originates from multiplying the standardized matrix $\mathcal{N}$ by the criterion weight coefficients ${\mathcal{W}}_{\mathcal{j}}$, according to Eq. (17). This paper defines the weight coefficients for each criterion via the entropy objective weighting technique from the preceding phase.

17 $${\mathcal{v}}_{\mathcal{i}\mathcal{j}}={\mathcal{n}}_{\mathcal{i}\mathcal{j}}\times {\mathcal{W}}_{\mathcal{j}}$$

*Step 5* Determine the utility values of each alternative corresponding to the AI and AAI solutions. From (6), we have

18 $$\begin{aligned}{\mathcal{K}}_{\mathcal{i}}^{+}=&\;{\mathrm{D}}_{\mathrm{NCSIVN}}(\mathrm{AI},\mathrm{AAI})\\=&\;\frac{2}{\sqrt{15\mathrm{n}}}\sqrt{\sum_{\mathrm{k}=1}^{\mathrm{n}}{\sum }_{\mathrm{M}\in \left\{\mathrm{T},\mathrm{I},\mathrm{F}\right\}}{({({\mathrm{C}}_{\mathrm{M}}}^{\left(\mathrm{k}\right)}\left(\mathrm{AI}\right)-{({\mathrm{C}}_{\mathrm{M}}}^{\left(\mathrm{k}\right)}\left(\left(\mathrm{AAI}\right)\right))}^{2}+{{({\mathrm{S}}_{\mathrm{M}}}^{\left(\mathrm{k}\right)}\left(\mathrm{AI}\right)-{({\mathrm{S}}_{\mathrm{M}}}^{\left(\mathrm{k}\right)}\left(\mathrm{AAI}\right))}^{2}})\end{aligned}$$

19 $$\begin{aligned}{\mathcal{K}}_{\mathcal{i}}^{-}=&\;{\mathrm{D}}_{\mathrm{NCSIVN}}(\mathrm{AI},\mathrm{AAI})\\=&\;\frac{2}{\sqrt{15\mathrm{n}}}\sqrt{\sum_{\mathrm{k}=1}^{\mathrm{n}}{\sum }_{\mathrm{M}\in \left\{\mathrm{T},\mathrm{I},\mathrm{F}\right\}}{({({\mathrm{C}}_{\mathrm{M}}}^{\left(\mathrm{k}\right)}\left(\mathrm{AI}\right)-{({\mathrm{C}}_{\mathrm{M}}}^{\left(\mathrm{k}\right)}\left(\mathrm{AAI}\right))}^{2}+{{({\mathrm{S}}_{\mathrm{M}}}^{\left(\mathrm{k}\right)}\left(\mathrm{AI}\right)-{({\mathrm{S}}_{\mathrm{M}}}^{\left(\mathrm{k}\right)}\left(\mathrm{AAI}\right))}^{2}}\end{aligned}$$

The normalized centroid spread interval-valued neutrosophic distance (NC-SIVNDM) measure for each alternative, corresponding to the IVN ideal $\left({\mathcal{K}}_{\mathcal{i}}^{+}\right)$ and anti-ideal $\left({\mathcal{K}}_{\mathcal{i}}^{-}\right)$ solutions, can be obtained via Eqs. (18) and (19) to derive the final utility scorecards.

*Step 6* The utility function of the alternatives $f\left({\mathcal{K}}_{\mathcal{i}}\right)$ can be obtained from Eq. (20). The utility function illustrates the compromise between the noticeable alternative and the ideal and anti-ideal solutions*.*

20 $$f\left({\mathcal{K}}_{\mathcal{i}}\right)=\frac{{\mathcal{K}}_{\mathcal{i}}^{+}+{\mathcal{K}}_{\mathcal{i}}^{-}}{1+\frac{1-f\left({\mathcal{K}}_{\mathcal{i}}^{+}\right)}{f\left({\mathcal{K}}_{\mathcal{i}}^{+}\right)}+\frac{1-f\left({\mathcal{K}}_{\mathcal{i}}^{-}\right)}{f\left({\mathcal{K}}_{\mathcal{i}}^{-}\right)}}$$

The utility functions associated with the ideal $f\left({\mathcal{K}}_{\mathcal{i}}^{+}\right)$ and anti-ideal $f\left({\mathcal{K}}_{\mathcal{i}}^{-}\right)$ solutions were established via Eqs. (21) and (22).

21 $$f\left({\mathcal{K}}_{\mathcal{i}}^{+}\right)=\frac{{\mathcal{K}}_{\mathcal{i}}^{-}}{{\mathcal{K}}_{\mathcal{i}}^{+}+{\mathcal{K}}_{\mathcal{i}}^{-}}$$

22 $$f\left({\mathcal{K}}_{\mathcal{i}}^{-}\right)=\frac{{\mathcal{K}}_{\mathcal{i}}^{+}}{{\mathcal{K}}_{\mathcal{i}}^{+}+{\mathcal{K}}_{\mathcal{i}}^{-}}$$

*Step 7* Rank the alternatives according to the final values of the utility function $f\left({\mathcal{K}}_{\mathcal{i}}\right)$.

The best alternative is the one closest to the ideal while simultaneously farthest from the anti-ideal reference point. An alternative with a greater utility function value is more desirable.

### Conversion of quantitative data into IVN scale

In this study, the analysis is deliberately restricted to the top ten hydrogen-leading countries based on data availability and relevance reported in the IEA global hydrogen review 2024. The quantitative indicators for these countries were converted to IVN representations using a structured, rule-based procedure. First, the collected quantitative data were normalized to the [0,1] interval to ensure comparability across countries. Subsequently, the normalized values were mapped into predefined linguistic categories using a fixed numerical scorecard, where values in the ranges 0.0–0.2, 0.2–0.4, 0.4–0.6, 0.6–0.8, and 0.8–1.0 correspond to the linguistic terms Very Low, Low, Medium, High, and Very High, respectively. Each linguistic term was then converted into its corresponding IVN number using an established linguistic-neutrosophic scale (Table 2). This conversion process does not rely on subjective expert judgment to assign linguistic values; instead, deterministic scorecard rules ensure objectivity, consistency, and reproducibility. No expert judgment was involved at any stage of the data conversion process. The mapping from normalized quantitative values to linguistic terms and corresponding IVN numbers was performed exclusively using predefined deterministic scorecard rules to ensure full objectivity, transparency, and reproducibility. To mitigate potential concerns about information loss, robustness and sensitivity tests were performed utilizing alternate weighting schemes and ranking methodologies, resulting in highly stable nation rankings. This verifies that the numerical-to-linguistic IVN transition maintains relative performance trends while improving interpretability in unpredictable conditions.

### Limitations related to secondary data utilization

The IEA global hydrogen review 2024 offers a comprehensive and internationally recognized dataset; however, the dependence on secondary data presents specific limitations that warrant acknowledgment. The reported indicators are aggregated at the national or regional level, potentially overlooking intra-country heterogeneity, regional disparities, and project-level operational performance. Secondly, various indicators such as projected capacity by 2030 and export-oriented capacity are anticipatory and contingent upon policy stability, investment actualization, and technological implementation, all of which are prone to uncertainty and potential modification over time. The temporal discrepancy between the swift advancement of hydrogen projects and the periodic publication of reports may result in time-lag effects, whereby recent developments are not promptly incorporated into the dataset.

The conversion of quantitative indicators into linguistic and IVN representations, despite being executed via deterministic and reproducible scorecard rules, can lead to information smoothing and diminished granularity, especially when countries display closely clustered numerical values. Consequently, due to the analysis being limited to countries with adequate and consistent data availability, the results may not be readily applicable to emerging or data-deficient regions. The identified limitations do not diminish the methodological validity of the proposed IVN-Entropy-NC-SIVNDM MARCOS framework; instead, they underscore the necessity for careful interpretation of results and encourage future research utilizing primary data, project-level indicators, and dynamic datasets. A consolidated discussion of these limitations and their implications is provided in the research limitations section.

## Results

In this section, the performance of the proposed IVN-Entropy-NC-SIVNDM MARCOS framework is evaluated using empirical data from the global hydrogen review 2024 and validated through comparative and sensitivity analyses.

### Case study

The present study utilizes data from the global hydrogen review 2024 published by the international energy agency (IEA), which provides verified and comprehensive insights into global trends in hydrogen production and development. The dataset includes ten prominent countries actively engaged in green hydrogen production, selected based on the availability, consistency, and reliability of quantitative data across all evaluation criteria. All the data were obtained from publicly accessible and reputable international energy statistics to ensure transparency and comparability. The selected countries are presented as alternatives in Table 3.

**Table 3:** List of selected green hydrogen-producing countries (alternatives) and primary project locations

S.no	Green hydrogen production countries	Symbol	Headquarters
1	China	${\mathcal{A}}_{1}$	Kuqa, Xinjiang
2	United States	${\mathcal{A}}_{2}$	Eastern Louisiana Hydrogen Complex
3	European Union (EU)	${\mathcal{A}}_{3}$	North Sea Region (Rotterdam Cluster)
4	Australia	${\mathcal{A}}_{4}$	Kalgoorlie, Western Australia
5	Japan	${\mathcal{A}}_{5}$	Tokyo Bay Region
6	Saudi Arabia	${\mathcal{A}}_{6}$	NEOM (Oxagon)
7	Chile	${\mathcal{A}}_{7}$	Magallanes Region
8	India	${\mathcal{A}}_{8}$	Paradip Port, Odisha
9	Brazil	${\mathcal{A}}_{9}$	Port of Pecém, Ceará
10	Canada	${\mathcal{A}}_{10}$	Alberta Industrial Heartland

(Data source: *IEA Global Hydrogen Review 2024*)

The evaluation criteria were chosen following an extensive examination of the literature and pertinent energy sustainability frameworks, as illustrated in Table 4. All criteria presented in Table 4 are quantitative in nature, enabling objective measurement and comparison across countries. The data corresponding to all evaluation criteria were sourced consistently from a single primary reference, namely the IEA global hydrogen review 2024, and were systematically gathered and analyzed in accordance with the established criteria for each chosen country.

**Table 4:** Evaluation criteria for assessing green hydrogen-producing countries

S.no	Criterion	Symbol	Description
1	Current Low-Emissions Hydrogen Production (million tonnes/year)	$${\mathcal{C}}_{1}$$	Indicates the present volume of hydrogen produced through renewable or low-emission sources, showing existing production capability
2	Announced Production Capacity by 2030 (million tonnes/year)	$${\mathcal{C}}_{2}$$	Represents future planned production capacity, reflecting national ambition and investment in hydrogen expansion
3	Installed Electrolyser Capacity (MW)	$${\mathcal{C}}_{3}$$	Denotes the operational electrolyser capacity currently installed for hydrogen generation
4	Announced Electrolyser Capacity by 2030 (MW)	$${\mathcal{C}}_{4}$$	Shows the planned electrolyser capacity for 2030, indicating scaling potential
5	Percentage of 2030 Announced Capacity with Final Investment Decision (FID)	$${\mathcal{C}}_{5}$$	Measures the maturity level of announced projects—the share that has reached firm investment status
6	Export-Oriented Capacity (million tonnes hydrogen equivalent/year)	$${\mathcal{C}}_{6}$$	Represents hydrogen production intended for export markets, reflecting international competitiveness and trade potential
7	Levelized Cost of Hydrogen (LCOH) Approx. ($/kg)	$${\mathcal{C}}_{7}$$	Average production cost per kilogram of hydrogen, capturing economic efficiency of production
8	Energy Consumption per kg Hydrogen Produced (kWh/kg)	$${\mathcal{C}}_{8}$$	Represents energy intensity of hydrogen production; lower consumption indicates higher process efficiency

(Data source: *IEA Global Hydrogen Review 2024*)

Among the eight selected criteria, ${\mathcal{C}}_{1}$ to ${\mathcal{C}}_{6}$ are identified as benefit-type criteria, where higher values indicate better performance, such as greater production capacity or investment maturity. Conversely, ${\mathcal{C}}_{7}$ and ${\mathcal{C}}_{8}$ are non-benefit-type criteria, where lower values represent better efficiency and cost-effectiveness. All the criteria are quantitative in nature, allowing for objective measurement and comparison across countries.

### Empirical results

#### Determination of criteria weights via the entropy method

The entropy weighting approach is utilized during this phase to determine the weights of significance with objective references. The initial IVN decision matrix, in which each alternative (${\mathcal{A}}_{\mathcal{i}}$) is evaluated against every criterion (${\mathcal{C}}_{\mathcal{j}}$) as an IVN number using the IVN scale from Table 2, is constructed based on the gathered information and is presented in Table 5.

**Table 5:** Preliminary IVN decision matrix

${\mathcal{A}}_{\mathcal{i}}/{\mathcal{C}}_{\mathcal{j}}$	${\mathcal{C}}_{1}$	${\mathcal{C}}_{2}$	${\mathcal{C}}_{3}$	${\mathcal{C}}_{4}$	${\mathcal{C}}_{5}$	${\mathcal{C}}_{6}$	${\mathcal{C}}_{7}$	${\mathcal{C}}_{8}$
${\mathcal{A}}_{1}$	([0.7,0.8], [0.2,0.3], [0.1,0.2])	([0.7,0.8], [0.2,0.3], [0.1,0.2])	([0.7,0.8], [0.2,0.3], [0.1,0.2])	([0.7,0.8], [0.2,0.3], [0.1,0.2])	([0.1,0.2], [0.7,0.8], [0.6,0.7])	([0.1,0.2], [0.7,0.8], [0.6,0.7])	([0.4,0.5], [0.5,0.6], [0.4,0.5])	([0.3,0.4], [0.6,0.7], [0.5,0.6])
${\mathcal{A}}_{2}$	([0.1,0.2], [0.7,0.8], [0.6,0.7])	([0.7,0.8], [0.2,0.3], [0.1,0.2])	([0.1,0.2], [0.7,0.8], [0.6,0.7])	([0.6,0.7], [0.4,0.5], [0.3,0.4])	([0.3,0.4], [0.6,0.7], [0.5,0.6])	([0.4,0.5], [0.5,0.6], [0.4,0.5])	([0.6,0.7], [0.4,0.5], [0.3,0.4])	([0.4,0.5], [0.5,0.6], [0.4,0.5])
${\mathcal{A}}_{3}$	([0.3,0.4], [0.6,0.7], [0.5,0.6])	([0.7,0.8], [0.2,0.3], [0.1,0.2])	([0.3,0.4], [0.6,0.7], [0.5,0.6])	([0.6,0.7], [0.4,0.5], [0.3,0.4])	([0.1,0.2], [0.7,0.8], [0.6,0.7])	([0.3,0.4], [0.6,0.7], [0.5,0.6])	([0.7,0.8], [0.2,0.3], [0.1,0.2])	([0.4,0.5], [0.5,0.6], [0.4,0.5])
${\mathcal{A}}_{4}$	([0.1,0.2], [0.7,0.8], [0.6,0.7])	([0.6,0.7], [0.4,0.5], [0.3,0.4])	([0.1,0.2], [0.7,0.8], [0.6,0.7])	([0.6,0.7], [0.4,0.5], [0.3,0.4])	([0.1,0.2], [0.7,0.8], [0.6,0.7])	([0.7,0.8], [0.2,0.3], [0.1,0.2])	([0.3,0.4], [0.6,0.7], [0.5,0.6])	([0.3,0.4], [0.6,0.7], [0.5,0.6])
${\mathcal{A}}_{5}$	([0.1,0.2], [0.7,0.8], [0.6,0.7])	([0.3,0.4], [0.6,0.7], [0.5,0.6])	([0.1,0.2], [0.7,0.8], [0.6,0.7])	([0.1,0.2], [0.7,0.8], [0.6,0.7])	([0.4,0.5], [0.5,0.6], [0.4,0.5])	([0.4,0.5], [0.5,0.6], [0.4,0.5])	([0.6,0.7], [0.4,0.5], [0.3,0.4])	([0.4,0.5], [0.5,0.6], [0.4,0.5])
${\mathcal{A}}_{6}$	([0.1,0.2], [0.7,0.8], [0.6,0.7])	([0.3,0.4], [0.6,0.7], [0.5,0.6])	([0.1,0.2], [0.7,0.8], [0.6,0.7])	([0.1,0.2], [0.7,0.8], [0.6,0.7])	([0.7,0.8], [0.2,0.3], [0.1,0.2])	([0.4,0.5], [0.5,0.6], [0.4,0.5])	([0.1,0.2], [0.7,0.8], [0.6,0.7])	([0.1,0.2], [0.7,0.8], [0.6,0.7])
${\mathcal{A}}_{7}$	([0.1,0.2], [0.7,0.8], [0.6,0.7])	([0.3,0.4], [0.6,0.7], [0.5,0.6])	([0.1,0.2], [0.7,0.8], [0.6,0.7])	([0.1,0.2], [0.7,0.8], [0.6,0.7])	([0.1,0.2], [0.7,0.8], [0.6,0.7])	([0.3,0.4], [0.6,0.7], [0.5,0.6])	([0.3,0.4], [0.6,0.7], [0.5,0.6])	([0.3,0.4], [0.6,0.7], [0.5,0.6])
${\mathcal{A}}_{8}$	([0.1,0.2], [0.7,0.8], [0.6,0.7])	([0.3,0.4], [0.6,0.7], [0.5,0.6])	([0.1,0.2], [0.7,0.8], [0.6,0.7])	([0.1,0.2], [0.7,0.8], [0.6,0.7])	([0.1,0.2], [0.7,0.8], [0.6,0.7])	([0.1,0.2], [0.7,0.8], [0.6,0.7])	([0.4,0.5], [0.5,0.6], [0.4,0.5])	([0.4,0.5], [0.5,0.6], [0.4,0.5])
${\mathcal{A}}_{9}$	([0.1,0.2], [0.7,0.8], [0.6,0.7])	([0.1,0.2], [0.7,0.8], [0.6,0.7])	([0.1,0.2], [0.7,0.8], [0.6,0.7])	([0.1,0.2], [0.7,0.8], [0.6,0.7])	([0.1,0.2], [0.7,0.8], [0.6,0.7])	([0.1,0.2], [0.7,0.8], [0.6,0.7])	([0.4,0.5], [0.5,0.6], [0.4,0.5])	([0.4,0.5], [0.5,0.6], [0.4,0.5])
${\mathcal{A}}_{10}$	([0.1,0.2], [0.7,0.8], [0.6,0.7])	([0.1,0.2], [0.7,0.8], [0.6,0.7])	([0.1,0.2], [0.7,0.8], [0.6,0.7])	([0.1,0.2], [0.7,0.8], [0.6,0.7])	([0.1,0.2], [0.7,0.8], [0.6,0.7])	([0.1,0.2], [0.7,0.8], [0.6,0.7])	([0.6,0.7], [0.4,0.5], [0.3,0.4])	([0.4,0.5], [0.5,0.6], [0.4,0.5])

The entropy values (${\mathcal{E}}_{\mathcal{j}}$) for each criterion (${\mathcal{C}}_{\mathcal{j}}$), illustrating their knowledge richness, along with the corresponding objective weights (${\mathcal{W}}_{\mathcal{j}}$) determined from the entropy approach, are combined, highlighting the relative significance of each criterion in the decision-making process, as presented in Table 6.

$${\mathcal{E}}_{\mathcal{j}} for {\mathcal{C}}_{1}=\frac{(1-{u}_{\mathcal{j}})}{(1+{u}_{\mathcal{j}})}=\frac{(1-0.60)}{(1+0.60)}=0.250$$

where

$$\begin{aligned}{u}_{\mathcal{j}}=&\;\frac{1}{32}\sum [\left|0.7-0.5\right|+\left|0.8-0.5\right|+\left|0.2-0.5\right|+\left|0.3-0.5\right|\\&+\left|0.5-0.3\right|+\left|0.5-0.2\right|+\left|0.1-0.5\right|+\left|0.2-0.5\right|=0.60\end{aligned}$$

**Table 6:** Entropy values and criteria weights

	${\mathcal{C}}_{1}$	${\mathcal{C}}_{2}$	${\mathcal{C}}_{3}$	${\mathcal{C}}_{4}$	${\mathcal{C}}_{5}$	${\mathcal{C}}_{6}$	${\mathcal{C}}_{7}$	${\mathcal{C}}_{8}$
Entropy value $\left({\mathcal{E}}_{\mathcal{j}}\right)$	0.250	0.350	0.250	0.317	0.290	0.410	0.531	0.624
Weight of Criteria $\left({\mathcal{W}}_{\mathcal{j}}\right)$	0.151	0.131	0.151	0.137	0.143	0.119	0.094	0.075

$${\mathcal{W}}_{\mathcal{j}} for {\mathcal{C}}_{1}=\frac{\left(1-0.250\right)}{\sum_{\mathcal{j}=1}^{8}\left((1-0.250)+(1-0.350)+\dots +(1-0.624)\right)}=0.151$$

**Fig. 2:**
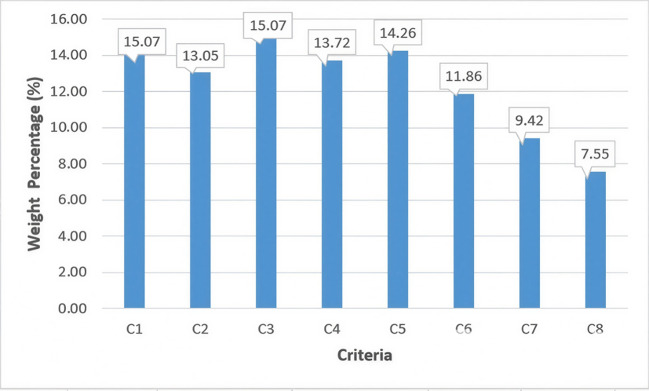
Weight percentage distributions of the green hydrogen evaluation criteria

The weights of all eight quantitative criteria are presented in Table 6. The relative significance of these criteria is illustrated in Fig. 2. The current low-emissions hydrogen production (Criteria 1) and installed electrolyser capacity (Criteria 3) have the highest influence levels, each at 15.07%, followed by the percentage of the capacity in 2030 that reached the FID (Criteria 5) at 14.26% and the announced electrolyser capacity by 2030 (Criteria 4) at 13.72%. These results indicate that hydrogen production performance and electrolyser development factors play a dominant role in the comprehensive evaluation of green hydrogen progress among countries. In Fig. 2, C1–C8 represent Criteria 1–8, respectively.

#### Ranking of alternatives via the NC-SIVNDM MARCOS model

A MARCOS approach is used to rank the alternatives in accordance with the suggested NC-SIVNDM MARCOS framework. This is done after the objective weights of the criteria have been determined. An ideal (AI) solution and an anti-ideal (AAI) solution are determined for each criterion via the process described below. The ideal solution corresponds to the highest possible value of a criterion, whereas the anti-ideal solution corresponds to the lowest possible value. Both solutions serve as reference points for evaluating all the alternatives.

The AI and AAI solutions, extracted from the preliminary IVN decision matrix (Table 5), are summarized in Table 7. AI symbolizes optimal performance, whereas AAI signifies the least favorable performance across all criteria, serving as reference points for calculating utility functions and ranking alternatives.

**Table 7:** AI and AAI solutions of the extended decision matrix (based on Table 5)

	${\mathcal{C}}_{1}$	${\mathcal{C}}_{2}$	${\mathcal{C}}_{3}$	${\mathcal{C}}_{4}$	${\mathcal{C}}_{5}$	${\mathcal{C}}_{6}$	${\mathcal{C}}_{7}$	${\mathcal{C}}_{8}$
AAI	([0.1,0.2], [0.7,0.8], [0.6,0.7])	([0.1,0.2], [0.7,0.8], [0.6,0.7])	([0.1,0.2], [0.7,0.8], [0.6,0.7])	([0.1,0.2], [0.7,0.8], [0.6,0.7])	([0.1,0.2], [0.7,0.8], [0.6,0.7])	([0.1,0.2], [0.7,0.8], [0.6,0.7])	([0.7,0.8], [0.2,0.3], [0.1,0.2])	([0.4,0.5], [0.5,0.6], [0.4,0.5])
AI	([0.7,0.8], [0.2,0.3], [0.1,0.2])	([0.7,0.8], [0.2,0.3], [0.1,0.2])	([0.7,0.8], [0.2,0.3], [0.1,0.2])	([0.7,0.8], [0.2,0.3], [0.1,0.2])	([0.7,0.8], [0.2,0.3], [0.1,0.2])	([0.7,0.8], [0.2,0.3], [0.1,0.2])	([0.1,0.2], [0.7,0.8], [0.6,0.7])	([0.1,0.2], [0.7,0.8], [0.6,0.7])

$$AAI=\left\{\begin{array}{c}min\left\{\left(\mathrm{0.7,0.8}\right), \left(\mathrm{0.1,0.2}\right),\dots , \left(\mathrm{0.1,0.2}\right)\right\}, max\left\{\left(\mathrm{0.2,0.3}\right), \left(\mathrm{0.7,0.8}\right),\dots ,\left(\mathrm{0.7,0.8}\right)\right\},\\ max\left\{\left(\mathrm{0.1,0.2}\right),\left(\mathrm{0.6,0.7}\right), \dots , \left(\mathrm{0.6,0.7}\right)\right\}\end{array}\right\}$$

$$AAI for {\mathcal{C}}_{1}=([\mathrm{0.1,0.2}], [\mathrm{0.7,0}.8], [\mathrm{0.6,0.7}])$$

$$AI=\left\{\begin{array}{c}max\left\{\left(\mathrm{0.7,0.8}\right), \left(\mathrm{0.1,0.2}\right),\dots , \left(\mathrm{0.1,0.2}\right)\right\}, min\left\{\left(\mathrm{0.2,0.3}\right), \left(\mathrm{0.7,0.8}\right),\dots ,\left(\mathrm{0.7,0.8}\right)\right\},\\ min\left\{\left(\mathrm{0.1,0.2}\right),\left(\mathrm{0.6,0.7}\right), \dots , \left(\mathrm{0.6,0.7}\right)\right\}\end{array}\right\}$$

$$AI for {\mathcal{C}}_{1}=([\mathrm{0.7,0.8}], [\mathrm{0.2,0.3}], [\mathrm{0.1,0.2}])$$

The normalized values of all alternatives, highlighting their relative performance for both beneficial and nonbeneficial criteria, are shown in Table 8.

**Table 8:** Normalized extended decision matrix

	${\mathcal{C}}_{1}$	${\mathcal{C}}_{2}$	${\mathcal{C}}_{3}$	${\mathcal{C}}_{4}$	${\mathcal{C}}_{5}$	${\mathcal{C}}_{6}$	${\mathcal{C}}_{7}$	${\mathcal{C}}_{8}$
**AAI**	([0.14,0.25], [3.50,2.67], [6.00,3.50])	([0.14,0.25], [3.50,2.67], [6.00,3.50])	([0.14,0.25], [3.50,2.67], [6.00,3.50])	([0.14,0.25], [3.50,2.67], [6.00,3.50])	([0.14,0.25], [3.50,2.67], [6.00,3.50])	([0.14,0.25], [3.50,2.67], [6.00,3.50])	([1.00,1.00], [1.00,1.00], [1.00, 1.00])	([1.00,1.00], [1.00,1.00], [1.00, 1.00])
${\mathcal{A}}_{1}$	([1.00,1.00], [1.00,1.00], [1.00, 1.00])	([1.00,1.00], [1.00,1.00], [1.00, 1.00])	([1.00,1.00], [1.00,1.00], [1.00, 1.00])	([1.00,1.00], [1.00,1.00], [1.00, 1.00])	([0.14,0.25], [3.50,2.67], [6.00,3.50])	([0.14,0.25], [3.50,2.67], [6.00,3.50])	([0.25,0.40], [1.40,1.33], [1.50, 1.40])	([0.33,0.50], [1.17,1.14], [1.20,1.17])
${\mathcal{A}}_{2}$	([0.14,0.25], [3.50,2.67], [6.00,3.50])	([1.00,1.00], [1.00,1.00], [1.00, 1.00])	([0.14,0.25], [3.50,2.67], [6.00,3.50])	([0.86,0.88], [2.00,1.67], [3.00, 2.00])	([0.43,0.50], [3.00,2.33], [5.00,3.00])	([0.57,0.63], [2.50,2.00], [4.00, 2.50])	([0.17,0.29], [1.75,1.60], [2.00,1.75])	([0.25,0.40], [1.40,1.33], [1.50,1.40])
${\mathcal{A}}_{3}$	([0.43,0.50], [3.00,2.33], [5.00,3.00])	([1.00,1.00], [1.00,1.00], [1.00, 1.00])	([0.43,0.50], [3.00,2.33], [5.00,3.00])	([0.86,0.88], [2.00,1.67], [3.00, 2.00])	([0.14,0.25], [3.50,2.67], [6.00,3.50])	([0.43,0.50], [3.00,2.33], [5.00,3.00])	([0.14,0.25], [3.50,2.67], [6.00,3.50])	([0.25,0.40], [1.40,1.33], [1.50,1.40])
${\mathcal{A}}_{4}$	([0.14,0.25], [3.50,2.67], [6.00,3.50])	([0.86,0.88], [2.00,1.67], [3.00,2.00])	([0.14,0.25], [3.50,2.67], [6.00,3.50])	([0.86,0.88], [2.00,1.67], [3.00, 2.00])	([0.14,0.25], [3.50,2.67], [6.00,3.50])	([1.00,1.00], [1.00,1.00], [1.00, 1.00])	([0.33,0.50], [1.17,1.14], [1.20,1.17])	([0.33,0.50], [1.17,1.14], [1.20,1.17])
${\mathcal{A}}_{5}$	([0.14,0.25], [3.50,2.67], [6.00,3.50])	([0.43,0.50], [3.00,2.33], [5.00,3.00])	([0.14,0.25], [3.50,2.67], [6.00,3.50])	([0.14,0.25], [3.50,2.67], [6.00,3.50])	([0.57,0.63], [2.50,2.00], [4.00, 2.50])	([0.57,0.63], [2.50,2.00], [4.00, 2.50])	([0.17,0.29], [1.75,1.60], [2.00,1.75])	([0.25,0.40], [1.40,1.33], [1.50,1.40])
${\mathcal{A}}_{6}$	([0.14,0.25], [3.50,2.67], [6.00,3.50])	([0.43,0.50], [3.00,2.33], [5.00,3.00])	([0.14,0.25], [3.50,2.67], [6.00,3.50])	([0.14,0.25], [3.50,2.67], [6.00,3.50])	([1.00,1.00], [1.00,1.00], [1.00, 1.00])	([0.57,0.63], [2.50,2.00], [4.00, 2.50])	([1.00,1.00], [1.00,1.00], [1.00, 1.00])	([1.00,1.00], [1.00,1.00], [1.00, 1.00])
${\mathcal{A}}_{7}$	([0.14,0.25], [3.50,2.67], [6.00,3.50])	([0.43,0.50], [3.00,2.33], [5.00,3.00])	([0.14,0.25], [3.50,2.67], [6.00,3.50])	([0.14,0.25], [3.50,2.67], [6.00,3.50])	([0.14,0.25], [3.50,2.67], [6.00,3.50])	([0.43,0.50], [3.00,2.33], [5.00,3.00])	([0.33,0.50], [1.17,1.14], [1.20,1.17])	([0.33,0.50], [1.17,1.14], [1.20,1.17])
${\mathcal{A}}_{8}$	([0.14,0.25], [3.50,2.67], [6.00,3.50])	([0.43,0.50], [3.00,2.33], [5.00,3.00])	([0.14,0.25], [3.50,2.67], [6.00,3.50])	([0.14,0.25], [3.50,2.67], [6.00,3.50])	([0.14,0.25], [3.50,2.67], [6.00,3.50])	([0.14,0.25], [3.50,2.67], [6.00,3.50])	([0.25,0.40], [1.40,1.33], [1.50,1.40])	([0.25,0.40], [1.40,1.33], [1.50,1.40])
${\mathcal{A}}_{9}$	([0.14,0.25], [3.50,2.67], [6.00,3.50])	([0.14,0.25], [3.50,2.67], [6.00,3.50])	([0.14,0.25], [3.50,2.67], [6.00,3.50])	([0.14,0.25], [3.50,2.67], [6.00,3.50])	([0.14,0.25], [3.50,2.67], [6.00,3.50])	([0.14,0.25], [3.50,2.67], [6.00,3.50])	([0.25,0.40], [1.40,1.33], [1.50,1.40])	([0.25,0.40], [1.40,1.33], [1.50,1.40])
${\mathcal{A}}_{10}$	([0.14,0.25], [3.50,2.67], [6.00,3.50])	([0.14,0.25], [3.50,2.67], [6.00,3.50])	([0.14,0.25], [3.50,2.67], [6.00,3.50])	([0.14,0.25], [3.50,2.67], [6.00,3.50])	([0.14,0.25], [3.50,2.67], [6.00,3.50])	([0.14,0.25], [3.50,2.67], [6.00,3.50])	([0.17,0.29], [1.75,1.60], [2.00,1.75])	([0.25,0.40], [1.40,1.33], [1.50,1.40])
**AI**	([1.00,1.00], [1.00,1.00], [1.00, 1.00])	([1.00,1.00], [1.00,1.00], [1.00, 1.00])	([1.00,1.00], [1.00,1.00], [1.00, 1.00])	([1.00,1.00], [1.00,1.00], [1.00, 1.00])	([1.00,1.00], [1.00,1.00], [1.00, 1.00])	([1.00,1.00], [1.00,1.00], [1.00, 1.00])	([0.14,0.25], [3.50,2.67], [6.00,3.50])	([0.25,0.40], [1.40,1.33], [1.50,1.40])

$${\mathcal{A}}_{1} for {\mathcal{C}}_{1}=\left(\left[\frac{0.7}{0.7},\frac{0.8}{0.8}\right],\left[\frac{0.2}{0.2},\frac{0.3}{0.3}\right],\left[\frac{0.1}{0.1},\frac{0.2}{0.2}\right]\right)=([\mathrm{1.00,1.00}], [\mathrm{1.00,1.00}], [1.00, 1.00])$$

The next step involves weighing the normalized matrix via Eq. (17) by multiplying all the values of the normalized matrix by the criterion quantities. The weighted normalized matrix is listed in Table 9.

**Table 9:** Weighted normalized extended decision matrix

	${\mathcal{C}}_{1}$	${\mathcal{C}}_{2}$	${\mathcal{C}}_{3}$	${\mathcal{C}}_{4}$	${\mathcal{C}}_{5}$	${\mathcal{C}}_{6}$	${\mathcal{C}}_{7}$	${\mathcal{C}}_{8}$
**AAI**	([0.02,0.04], [0.53,0.40], [0.90,0.53])	([0.02,0.03], [0.46,0.35], [0.78,0.46])	([0.02,0.04], [0.53,0.40], [0.90,0.53])	([0.02,0.03], [0.48,0.37], [0.82,0.48])	([0.02,0.04], [0.50,0.38], [0.86,0.50])	([0.02,0.03], [0.42,0.32], [0.71,0.42])	([0.09,0.09], [0.09,0.09], [0.09,0.09])	([0.08,0.08], [0.08,0.08], [0.08,0.08])
${\mathcal{A}}_{1}$	([0.15,0.15], [0.15,0.15], [0.15,0.15])	([0.13,0.13], [0.13,0.13], [0.13,0.13])	([0.15,0.15], [0.15,0.15], [0.15,0.15])	([0.14,0.14], [0.14,0.14], [0.14,0.14])	([0.02,0.04], [0.50,0.38], [0.86,0.50])	([0.02,0.03], [0.42,0.32], [0.71,0.42])	([0.02,0.04], [0.13,0.13], [0.14,0.13])	([0.03,0.04], [0.09,0.09], [0.09,0.09])
${\mathcal{A}}_{2}$	([0.02,0.04], [0.53,0.40], [0.90,0.53])	([0.13,0.13], [0.13,0.13], [0.13,0.13])	([0.02,0.04], [0.53,0.40], [0.90,0.53])	([0.12,0.12], [0.27,0.23], [0.41,0.27])	([0.06,0.07], [0.43,0.33], [0.71,0.43])	([0.07,0.07], [0.30,0.24], [0.47,0.30])	([0.02,0.03], [0.16,0.15], [0.19,0.16])	([0.02,0.03], [0.11,0.10], [0.11,0.11])
${\mathcal{A}}_{3}$	([0.06,0.08], [0.45,0.35], [0.75,0.45])	([0.13,0.13], [0.13,0.13], [0.13,0.13])	([0.06,0.08], [0.45,0.35], [0.75,0.45])	([0.12,0.12], [0.27,0.23], [0.41,0.27])	([0.02,0.04], [0.50,0.38], [0.86,0.50])	([0.05,0.06], [0.36,0.28], [0.59,0.36])	([0.01,0.02], [0.33,0.25], [0.57,0.33])	([0.02,0.03], [0.11,0.10], [0.11,0.11])
${\mathcal{A}}_{4}$	([0.02,0.04], [0.53,0.40], [0.90,0.53])	([0.11,0.11], [0.26,0.22], [0.39,0.26])	([0.02,0.04], [0.53,0.40], [0.90,0.53])	([0.12,0.12], [0.27,0.23], [0.41,0.27])	([0.02,0.04], [0.50,0.38], [0.86,0.50])	([0.12,0.12], [0.12,0.12], [0.12,0.12])	([0.03,0.05], [0.11,0.11], [0.11,0.11])	(0.03,0.04], [0.09,0.09], [0.09,0.09])
${\mathcal{A}}_{5}$	([0.02,0.04], [0.53,0.40], [0.90,0.53])	([0.06,0.07], [0.39,0.30], [0.65,0.39])	([0.02,0.04], [0.53,0.40], [0.90,0.53])	([0.02,0.03], [0.48,0.37], [0.82,0.48])	([0.08,0.09], [0.36,0.29], [0.57,0.36])	([0.07,0.07], [0.30,0.24], [0.47,0.30])	([0.02,0.03], [0.16,0.15], [0.19,0.16])	([0.02,0.03], [0.11,0.10], [0.11,0.11])
${\mathcal{A}}_{6}$	([0.02,0.04], [0.53,0.40], [0.90,0.53])	([0.06,0.07], [0.39,0.30], [0.65,0.39])	([0.02,0.04], [0.53,0.40], [0.90,0.53])	([0.02,0.03], [0.48,0.37], [0.82,0.48])	([0.14,0.14], [0.14,0.14], [0.14,0.14])	([0.07,0.07], [0.30,0.24], [0.47,0.30])	([0.09,0.09], [0.09,0.09], [0.09,0.09])	([0.08,0.08], [0.08,0.08], [0.08,0.08])
${\mathcal{A}}_{7}$	([0.02,0.04], [0.53,0.40], [0.90,0.53])	([0.06,0.07], [0.39,0.30], [0.65,0.39])	([0.02,0.04], [0.53,0.40], [0.90,0.53])	([0.02,0.03], [0.48,0.37], [0.82,0.48])	([0.02,0.04], [0.50,0.38], [0.86,0.50])	([0.05,0.06], [0.36,0.28], [0.59,0.36])	([0.03,0.05], [0.11,0.11], [0.11,0.11])	([0.03,0.04], [0.09,0.09], [0.09,0.09])
${\mathcal{A}}_{8}$	([0.02,0.04], [0.53,0.40], [0.90,0.53])	([0.06,0.07], [0.39,0.30], [0.65,0.39])	([0.02,0.04], [0.53,0.40], [0.90,0.53])	([0.02,0.03], [0.48,0.37], [0.82,0.48])	([0.02,0.04], [0.50,0.38], [0.86,0.50])	([0.02,0.03], [0.42,0.32], [0.71,0.42])	([0.02,0.04], [0.13,0.13], [0.14,0.13])	([0.02,0.03], [0.11,0.10], [0.11,0.11])
${\mathcal{A}}_{9}$	([0.02,0.04], [0.53,0.40], [0.90,0.53])	([0.02,0.03], [0.46,0.35], [0.78,0.46])	([0.02,0.04], [0.53,0.40], [0.90,0.53])	([0.02,0.03], [0.48,0.37], [0.82,0.48])	([0.02,0.04], [0.50,0.38], [0.86,0.50])	([0.02,0.03], [0.42,0.32], [0.71,0.42])	([0.02,0.04], [0.13,0.13], [0.14,0.13])	([0.02,0.03], [0.11,0.10], [0.11,0.11])
${\mathcal{A}}_{10}$	([0.02,0.04], [0.53,0.40], [0.90,0.53])	([0.02,0.03], [0.46,0.35], [0.78,0.46])	([0.02,0.04], [0.53,0.40], [0.90,0.53])	([0.02,0.03], [0.48,0.37], [0.82,0.48])	([0.02,0.04], [0.50,0.38], [0.86,0.50])	([0.02,0.03], [0.42,0.32], [0.71,0.42])	([0.02,0.03], [0.16,0.15], [0.19,0.16])	([0.02,0.03], [0.11,0.10], [0.11,0.11])
**AI**	([0.15,0.15], [0.15,0.15], [0.15,0.15])	([0.13,0.13], [0.13,0.13], [0.13,0.13])	([0.15,0.15], [0.15,0.15], [0.15,0.15])	([0.14,0.14], [0.14,0.14], [0.14,0.14])	([0.14,0.14], [0.14,0.14], [0.14,0.14])	([0.12,0.12], [0.12,0.12], [0.12,0.12])	([0.01,0.02], [0.33,0.25], [0.57,0.33])	([0.02,0.03], [0.11,0.10], [0.11,0.11])

$${\mathcal{A}}_{1} for {\mathcal{C}}_{1}=\left(\left[0.151*\mathrm{1,0.151}*1\right], \left[0.151*\mathrm{1,0.151}*1\right],\left[0.151*\mathrm{1,0.151}*1\right]\right)$$

$${\mathcal{A}}_{1} for {\mathcal{C}}_{1}=([\mathrm{0.15,0.15}], [\mathrm{0.15,0.15}], [\mathrm{0.15,0.15}])$$

The utility values of each alternative were determined using the NC-SIVNDM MARCOS, which illustrates their proximity to the ideal (AI) and anti-ideal (AAI) solutions, as displayed in Table 10.

**Table 10:** Utility values of alternatives with respect to AI and AAI solutions

	${\mathcal{C}}_{1}$	${\mathcal{C}}_{2}$	${\mathcal{C}}_{3}$	${\mathcal{C}}_{4}$	${\mathcal{C}}_{5}$	${\mathcal{C}}_{6}$	${\mathcal{C}}_{7}$	${\mathcal{C}}_{8}$
**AAI**	([0.03,0.01], [0.46,− 0.06], [0.72,− 0.19])	([0.03,0.01], [0.40,− 0.05], [0.62,− 0.16])	([0.03,0.01], [0.46,− 0.06], [0.72,− 0.19])	([0.03,0.01], [0.42,− 0.06], [0.65,− 0.17])	([0.03,0.01], [0.44,− 0.06], [0.68,− 0.18])	([0.02,0.01], [0.37,− 0.05], [0.56,− 0.15])	([0.09,0.00], [0.09,0.00], [0.09,0.00])	([0.08,0.00], [0.08,0.00], [0.08,0.00])
${\mathcal{A}}_{1}$	([0.15,0.00], [0.15,0.00], [0.15,0.00])	([0.13,0.00], [0.13,0.00], [0.13,0.00])	([0.15,0.00], [0.15,0.00], [0.15,0.00])	([0.14,0.00], [0.14,0.00], [0.14,0.00])	([0.03,0.01], [0.44,− 0.06], [0.68,− 0.18])	([0.02,0.01], [0.37,− 0.05], [0.56,− 0.15])	([0.03,0.01], [0.13,0.00], [0.14,0.00])	([0.03,0.01], [0.09,0.00], [0.09,0.00])
${\mathcal{A}}_{2}$	([0.03,0.01], [0.46,− 0.06], [0.72,− 0.19])	([0.13,0.00], [0.13,0.00], [0.13,0.00])	([0.03,0.01], [0.46,− 0.06], [0.72,− 0.19])	([0.12,0.00], [0.25,− 0.02], [0.34,− 0.07])	([0.07,0.01], [0.38,− 0.05], [0.57,− 0.14])	([0.07,0.00], [0.27,− 0.03], [0.39,− 0.09])	([0.02,0.01], [0.16,− 0.01], [0.18,− 0.01])	([0.02,0.01], [0.10,0.00], [0.11,0.00])
${\mathcal{A}}_{3}$	([0.07,0.01], [0.40,− 0.05], [0.60,− 0.15])	([0.13,0.00], [0.13,0.00], [0.13,0.00])	([0.07,0.01], [0.40,− 0.05], [0.60,− 0.15])	([0.12,0.00], [0.25,− 0.02], [0.34,− 0.07])	([0.03,0.01], [0.44,− 0.06], [0.68,− 0.18])	([0.06,0.00], [0.32,− 0.04], [0.47,− 0.12]	([0.02,0.01], [0.29,− 0.04], [0.45,− 0.12])	([0.02,0.01], [0.10,0.00], [0.11,0.00])
${\mathcal{A}}_{4}$	([0.03,0.01], [0.46,− 0.06], [0.72,− 0.19])	([0.11,0.00], [0.24,− 0.02], [0.33,− 0.07])	([0.03,0.01], [0.46,− 0.06], [0.72,− 0.19])	([0.12,0.00], [0.25,− 0.02], [0.34,− 0.07])	([0.03,0.01], [0.44,− 0.06], [0.68,− 0.18])	([0.12,0.00], [0.12,0.00], [0.12,0.00])	([0.04,0.01], [0.11,0.00], [0.11,0.00])	([0.03,0.01], [0.09,0.00], [0.09,0.00])
${\mathcal{A}}_{5}$	([0.03,0.01], [0.46,− 0.06], [0.72,− 0.19])	([0.06,0.00], [0.35,− 0.04], [0.52,− 0.13])	([0.03,0.01], [0.46,− 0.06], [0.72,− 0.19])	([0.03,0.01], [0.42,− 0.06], [0.65,− 0.17])	([0.09,0.00], [0.32,− 0.04], [0.46,− 0.11])	([0.07,0.00], [0.27,− 0.03], [0.39,− 0.09])	([0.02,0.01], [0.16,− 0.01], [0.18,− 0.01])	([0.02,0.01], [0.10,0.00], [0.11,0.00])
${\mathcal{A}}_{6}$	([0.03,0.01], [0.46,− 0.06], [0.72,− 0.19])	([0.06,0.00], [0.35,− 0.04], [0.52,− 0.13])	([0.03,0.01], [0.46,− 0.06], [0.72,− 0.19])	([0.03,0.01], [0.42,− 0.06], [0.65,− 0.17])	([0.14,0.00], [0.14,0.00], [0.14,0.00])	([0.07,0.00], [0.27,− 0.03], [0.39,− 0.09])	([0.09,0.00], [0.09,0.00], [0.09,0.00])	([0.08,0.00], [0.08,0.00], [0.08,0.00])
${\mathcal{A}}_{7}$	([0.03,0.01], [0.46,− 0.06], [0.72,− 0.19])	([0.06,0.00], [0.35,− 0.04], [0.52,− 0.13])	([0.03,0.01], [0.46,− 0.06], [0.72,− 0.19])	([0.03,0.01], [0.42,− 0.06], [0.65,− 0.17])	([0.03,0.01], [0.44,− 0.06], [0.68,− 0.18])	([0.06,0.00], [0.32,− 0.04], [0.47,− 0.12]	([0.04,0.01], [0.11,0.00], [0.11,0.00])	([0.03,0.01], [0.09,0.00], [0.09,0.00])
${\mathcal{A}}_{8}$	([0.03,0.01], [0.46,− 0.06], [0.72,− 0.19])	([0.06,0.00], [0.35,− 0.04], [0.52,− 0.13])	([0.03,0.01], [0.46,− 0.06], [0.72,− 0.19])	([0.03,0.01], [0.42,− 0.06], [0.65,− 0.17])	([0.03,0.01], [0.44,− 0.06], [0.68,− 0.18])	([0.02,0.01], [0.37,− 0.05], [0.56,− 0.15])	([0.03,0.01], [0.13,0.00], [0.14,0.00])	([0.02,0.01], [0.10,0.00], [0.11,0.00])
${\mathcal{A}}_{9}$	([0.03,0.01], [0.46,− 0.06], [0.72,− 0.19])	([0.03,0.01], [0.40,− 0.05], [0.62,− 0.16])	([0.03,0.01], [0.46,− 0.06], [0.72,− 0.19])	([0.03,0.01], [0.42,− 0.06], [0.65,− 0.17])	([0.03,0.01], [0.44,− 0.06], [0.68,− 0.18])	([0.02,0.01], [0.37,− 0.05], [0.56,− 0.15])	([0.03,0.01], [0.13,0.00], [0.14,0.00])	([0.02,0.01], [0.10,0.00], [0.11,0.00])
${\mathcal{A}}_{10}$	([0.03,0.01], [0.46,− 0.06], [0.72,− 0.19])	([0.03,0.01], [0.40,− 0.05], [0.62,− 0.16])	([0.03,0.01], [0.46,− 0.06], [0.72,− 0.19])	([0.03,0.01], [0.42,− 0.06], [0.65,− 0.17])	([0.03,0.01], [0.44,− 0.06], [0.68,− 0.18])	([0.02,0.01], [0.37,− 0.05], [0.56,− 0.15])	([0.02,0.01], [0.16,− 0.01], [0.18,− 0.01])	([0.02,0.01], [0.10,0.00], [0.11,0.00])
**AI**	([0.15,0.00], [0.15,0.00], [0.15,0.00])	([0.13,0.00], [0.13,0.00], [0.13,0.00])	([0.15,0.00], [0.15,0.00], [0.15,0.00])	([0.14,0.00], [0.14,0.00], [0.14,0.00])	([0.14,0.00], [0.14,0.00], [0.14,0.00])	([0.12,0.00], [0.12,0.00], [0.12,0.00])	([0.02,0.01], [0.29,− 0.04], [0.45,− 0.12])	([0.02,0.01], [0.10,0.00], [0.11,0.00])

$${\mathcal{A}}_{1} for {\mathcal{C}}_{1}=\left(\left[\frac{0.15+0.15}{2}\right], \left[\frac{0.15+0.15}{2}\right], \left[\frac{0.15+0.15}{2}\right]\right)=[\mathrm{0.15,0.00}], [\mathrm{0.15,0.00}], [\mathrm{0.15,0.00}])$$

The determined utility function values $f\left({\mathcal{K}}_{\mathcal{i}}\right)$ for all alternatives, obtained from Eqs. (18–22) based on their proximity to the ideal and anti-ideal solutions, are detailed in Table 11. Higher $f\left({\mathcal{K}}_{\mathcal{i}}\right)$ values indicate alternatives that are nearer to the ideal solution, serving as the foundation for the ultimate ranking and selection of the best alternative.

**Table 11:** Final utility function values and ranking of alternatives

Alternatives	AI $({\mathcal{K}}_{\mathcal{i}}^{+})$	AAI (${\mathcal{K}}_{\mathcal{i}}^{-}$)	${\boldsymbol{f}}\left({\mathcal{K}}_{\mathcal{i}}^{+}\right)$	${\boldsymbol{f}}\left({\mathcal{K}}_{\mathcal{i}}^{-}\right)$	${\boldsymbol{f}}\left({\mathcal{K}}_{\mathcal{i}}\right)$	Rank
${\mathcal{A}}_{1}$	0.169	0.238	0.415	0.584	0.130	1
${\mathcal{A}}_{2}$	0.222	0.139	0.615	0.385	0.112	4
${\mathcal{A}}_{3}$	0.206	0.157	0.567	0.433	0.118	2
${\mathcal{A}}_{4}$	0.235	0.138	0.630	0.370	0.113	3
${\mathcal{A}}_{5}$	0.253	0.072	0.779	0.221	0.067	6
${\mathcal{A}}_{6}$	0.249	0.128	0.661	0.339	0.109	5
${\mathcal{A}}_{7}$	0.279	0.033	0.894	0.106	0.032	7
${\mathcal{A}}_{8}$	0.284	0.030	0.906	0.094	0.029	8
${\mathcal{A}}_{9}$	0.291	0.020	0.935	0.064	0.020	10
${\mathcal{A}}_{10}$	0.289	0.027	0.915	0.085	0.027	9

$${\mathcal{A}}_{1}=\frac{2}{\sqrt{15\times 8}}\sqrt{\left\{{\left(0.15-0.15\right)}^{2}+{\left(0.0-0.0\right)}^{2}+{\left(0.15-0.15\right)}^{2}+{\left(0.0-0.0\right)}^{2}+\dots +{\left(0.0-0.0\right)}^{2}\right\}}$$

$${\mathcal{A}}_{1} for {\mathcal{K}}_{\mathcal{i}}^{+}=0.1690$$

$${\mathcal{A}}_{1}=\frac{2}{\sqrt{15\times 8}}\sqrt{\left\{{\left(0.15-0.3\right)}^{2}+{\left(0.0-0.01\right)}^{2}+{\left(0.15-0.46\right)}^{2}+{\left(0.0-(-0.06)\right)}^{2}+\dots +{\left(0.0-0.0\right)}^{2}\right\}}$$

$${\mathcal{A}}_{1} for {\mathcal{K}}_{\mathcal{i}}^{-}=0.238$$

$${\mathcal{A}}_{1} for f\left({\mathcal{K}}_{\mathcal{i}}^{+}\right)=\frac{0.169}{\left(0.169+0.238\right)}=0.415$$

$${\mathcal{A}}_{1} for f\left({\mathcal{K}}_{\mathcal{i}}^{-}\right)=\frac{0.238}{\left(0.169+0.238\right)}=0.584$$

$${\mathcal{A}}_{1} for f\left({\mathcal{K}}_{\mathcal{i}}^{-}\right)=\frac{\left(0.169+0.238\right)}{\left\{1+\left(\frac{1-0.415}{0.415}\right)+\left(\frac{1-0.585}{0.584}\right)\right\}}=0.131$$

**Fig. 3:**
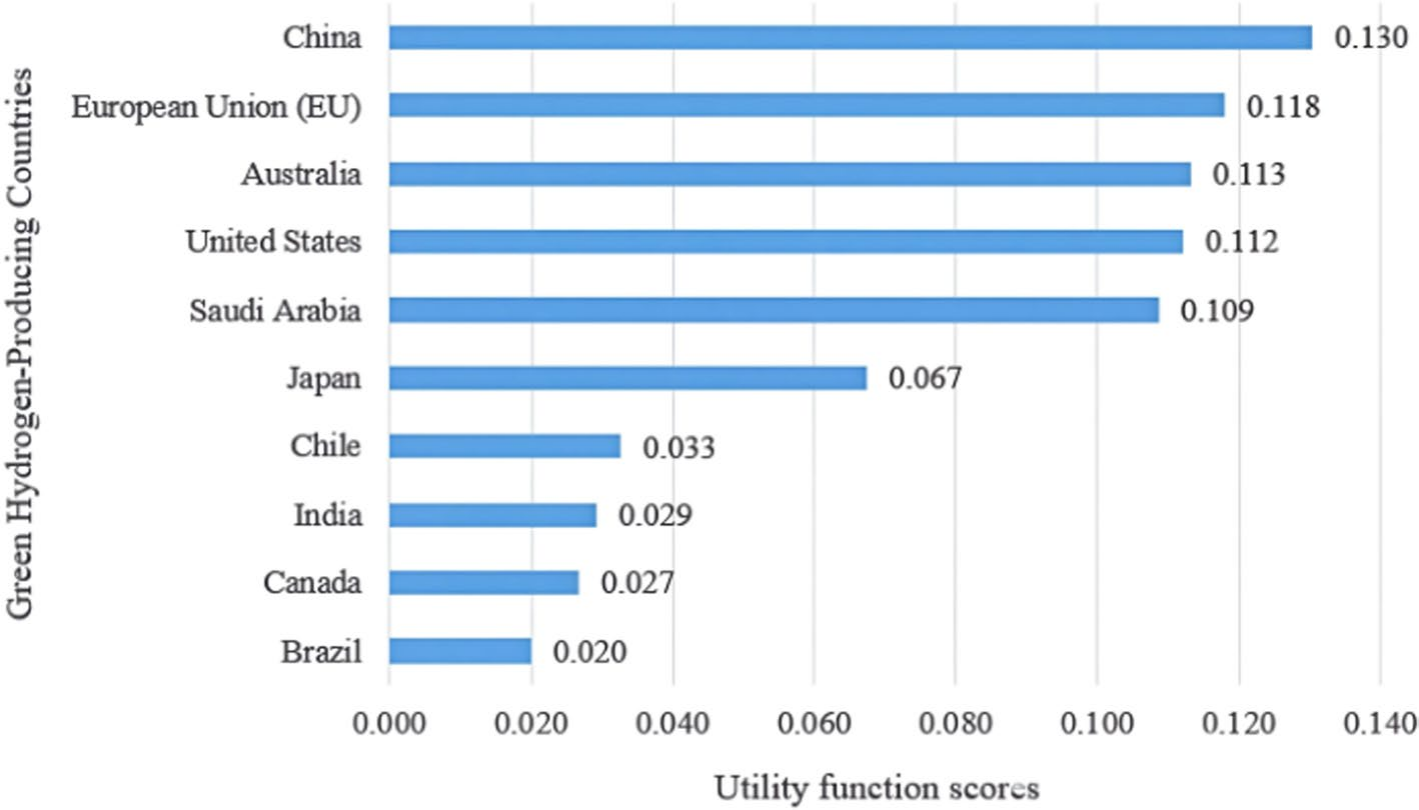
Final ranking of countries by IVN-Entropy-NC-SIVNDM MARCOS utility score

**Fig. 4:**
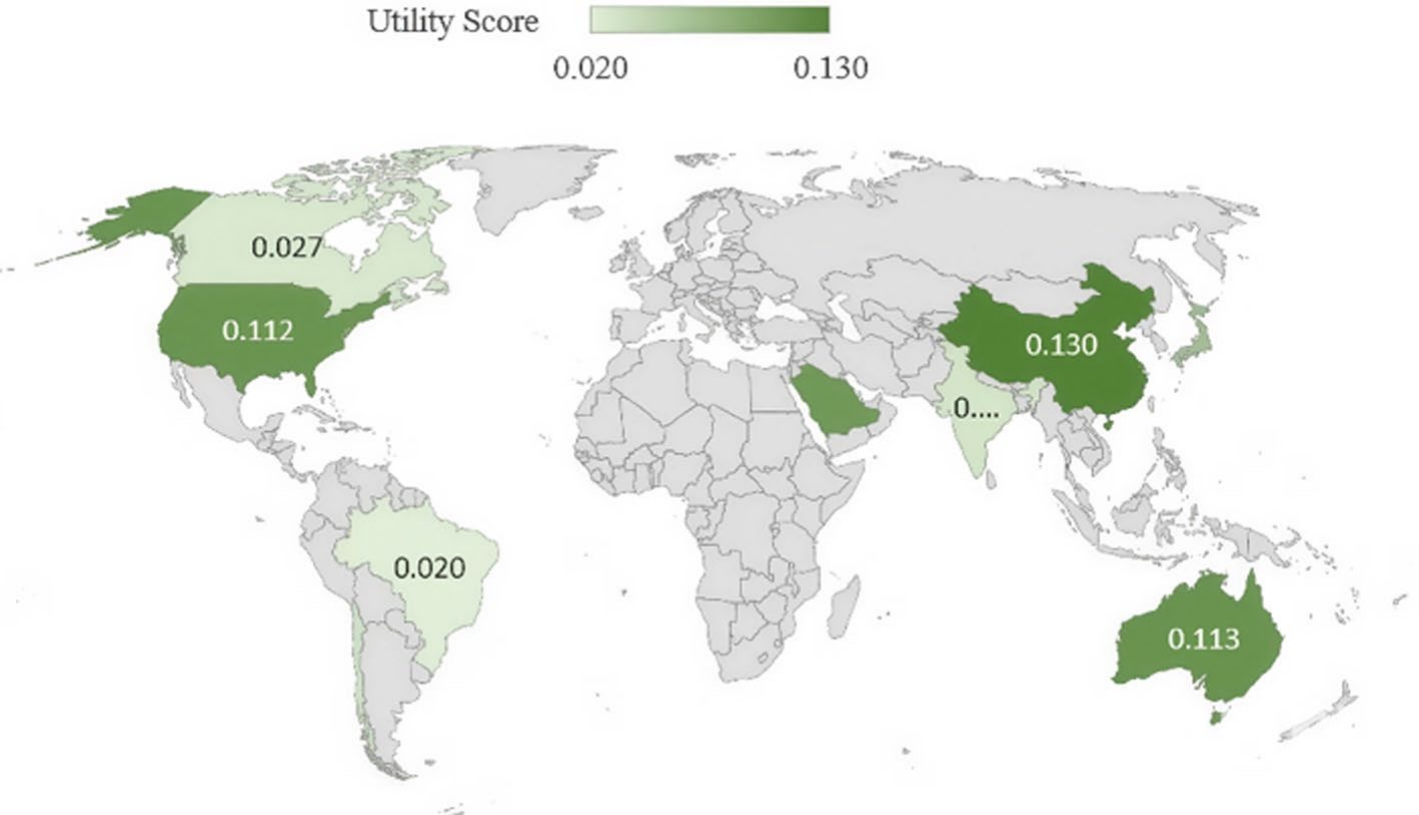
Geographical area map of the top-ranked green hydrogen-producing countries

The final utility scores $f\left({\mathcal{K}}_{\mathcal{i}}\right)$ for each country were computed via Eqs. (20–22). A higher $f\left({\mathcal{K}}_{\mathcal{i}}\right)$ indicates closer proximity to the ideal solution. The results of this research reveal that China ranks first, followed by the European Union in second place, and Australia in third, regarding their performance in green hydrogen production. The utility function scores recorded are 0.130, 0.118, and 0.113, underscoring China's prominent role in the global green hydrogen sector. Figure 3 presents the conclusive ranking of countries that produce green hydrogen, as established by the IVN-Entropy-Proposed NC-SIVNDM MARCOS model, and the corresponding area map is presented in Fig. 4.

China, the EU, and Australia ranked highest, reflecting their advanced electrolyser deployment and production capacity, whereas India and Brazil occupy mid-range positions.

### Results validation

#### Sensitivity and correlation analysis

A sensitivity analysis was conducted to evaluate the robustness and consistency of the proposed IVN-Entropy-NC-SIVNDM MARCOS framework. The analysis examined how changes in criterion weights affect the final rankings, thereby assessing the model’s robustness and stability. This analysis involved comparing outcomes derived from alternative weighting schemes IVN-AHP [69, 72], IVN-Equal [78], and IVN-CRITIC [79] with those determined via the proposed IVN-Entropy approach. All the models employed an identical set of eight evaluation criteria across ten countries that produce green hydrogen. This analysis aimed to assess the impact of various weighting mechanisms on the final rankings of alternatives. The IVN-AHP method is characterized by a subjective, expert-driven approach, whereas the IVN-Equal model posits equal significance for all criteria. The IVN-CRITIC and IVN-Entropy methods are entirely objective and are based on statistical variability and information entropy, respectively.

The comparative results indicate that both the top-ranked and mid-ranked alternatives exhibit significant stability across all weighting schemes, confirming the robustness of the proposed framework. The top three countries consistently maintained the same positions, demonstrating that variations in weighting methods have minimal influence on the ranking results. Although the United States remains within the upper tier, minor rank shifts were observed under alternative weighting scenarios. Minor differences were observed only in the lower-ranked alternatives (ranked 7–9), corresponding to slight variations in their performance scores. Importantly, while four weighting schemes, i.e., AHP, equal weight, CRITIC, and entropy (proposed), were employed to determine the criterion weights, the final rankings for all were computed via the common NC-SIVNDM MARCOS method. The comparative ranking results of the ten green hydrogen–producing countries under these weighting schemes are summarized in Table 12.

**Table 12:** Comparative ranking of alternatives under different weighting schemes in the IVN-based NC-SIVNDM MARCOS framework

Countries	AHP		Equal weight		CRICTIC		Entropy (Proposed)	
	Value	Rank	Value	Rank	Value	Rank	Value	Rank
China	0.097	1	0.125	1	0.131	1	0.131	1
United States	0.084	4	0.106	4	0.103	5	0.112	4
European Union	0.090	2	0.115	2	0.120	2	0.118	2
Australia	0.087	3	0.110	3	0.109	3	0.113	3
Japan	0.054	6	0.068	6	0.070	6	0.067	6
Saudi Arabia	0.074	5	0.098	5	0.106	4	0.109	5
Chile	0.030	7	0.037	7	0.034	8	0.033	7
India	0.029	9	0.036	9	0.034	9	0.029	8
Brazil	0.023	10	0.029	10	0.028	10	0.020	10
Canada	0.029	8	0.036	8	0.039	7	0.027	9

**Fig. 5:**
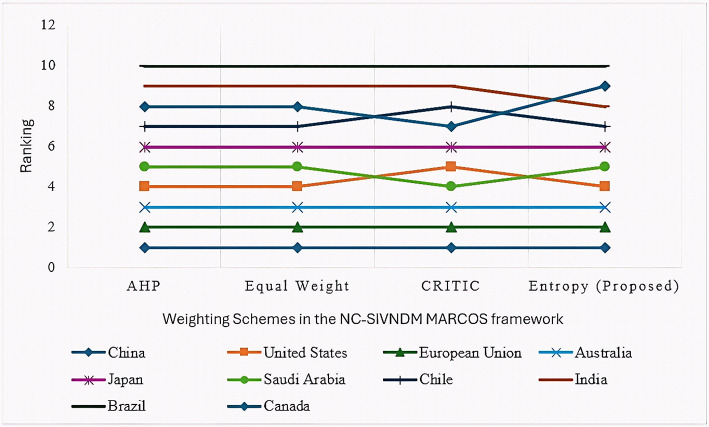
Ranking variation of green hydrogen-producing countries across four weighting schemes (AHP, equal weight, CRITIC, and entropy) via the common NC-SIVNDM MARCOS method

A comparison of the ranking results of countries that produce green hydrogen is presented in Fig. 5, which uses four MARCOS models that are based on IVN. Both the dependability and robustness of the suggested IVN-Entropy-NC-SIVNDM MARCOS strategy are confirmed by the fact that China, the European Union, and Australia have consistently ranked at the top of the respective rankings.

The reliability of the ranking outcomes was further evaluated via Spearman’s rank correlation coefficient $\left(\rho \right)$ across four weighting-based MARCOS variants. The proposed IVN-Entropy-NC-SIVNDM MARCOS model shows very high correlations with alternative weighting schemes, such as $\rho$ = 0.988 with IVN-AHP, ρ = 0.988 with IVN-Equal Weight, and ρ = 0.952 with IVN-CRITIC weighting. These strong positive correlations indicate that the ranking patterns are nearly identical across all weighting approaches, confirming the stability, consistency, and robustness of the proposed model in evaluating green hydrogen–producing countries. The robustness of the proposed framework was verified by comparing country rankings obtained using different weighting methods. The strong positive correlations ($\rho$ > 0.95) shown in Table 13 confirm the high consistency and stability of the IVN-Entropy-NC-SIVNDM MARCOS model.

**Table 13:** Spearman’s rank correlation $\left(\rho \right)$ among IVN-based NC-SIVNDM MARCOS models

Weighting method	AHP	Equal	CRITIC	Entropy (Proposed)
AHP	1.000	1.000	0.976	0.988
Equal weight	1.000	1.000	0.976	0.988
CRITIC	0.976	0.976	1.000	0.952
Entropy (Proposed)	0.988	0.988	0.952	1.000

All the models are NC-SIVNDM MARCOS variants distinguished by their weighting schemes. High correlation values (ρ > 0.95) indicate strong consistency and robustness among all the approaches.

### Comparative and correlation analysis of IVN MCDM methods

**Fig. 6:**
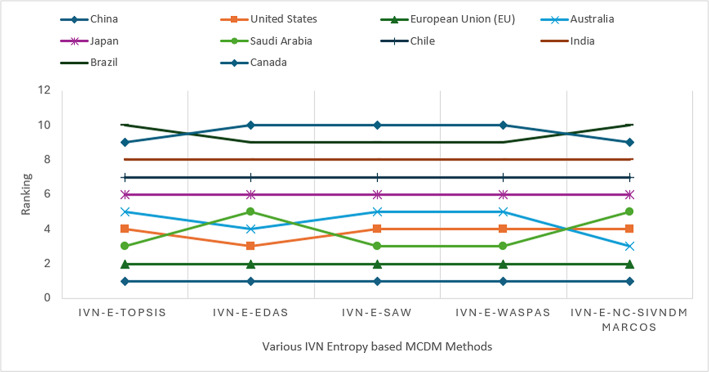
Comparative ranking consistency of countries across IVN-based MCDM methods

To assess the stability and dependability of the proposed IVN-Entropy-NC-SIVNDM MARCOS model, four alternative IVN-based MCDM techniques, i.e., IVN-TOPSIS [72, 80], IVN-EDAS [81, 82], IVN-SAW [83], and IVN-WASPAS [84, 85], were employed for comparison. In all the scenarios, the same criterion weights derived from the IVN-Entropy method were applied to ensure consistency in the weighting process, while the ranking of alternatives was determined via each MCDM approach. This setup enables a rigorous comparison of ranking performance under uniform weighting conditions. Figure 6 depicts the comparative results, and Table 14 presents the ranking outcomes of the ten green hydrogen-producing countries obtained from these IVN-based models via common IVN-entropy weights.

**Table 14:** Comparative rankings of countries under different IVN-based MCDM methods using common IVN-Entropy weights

Countries	TOPSIS		EDAS		SAW		WASPAS		NC-SIVNDC MARCOS (Proposed)	
	Value	Rank	Value	Rank	Value	Rank	Value	Rank	Value	Rank
China	0.600	1	0.849	1	1.063	1	0.688	1	0.131	1
United States	0.360	4	0.451	3	0.568	4	0.507	4	0.118	4
European Union	0.411	2	0.495	2	0.688	2	0.554	3	0.113	2
Australia	0.334	5	0.443	4	0.563	5	0.493	5	0.112	3
Japan	0.290	6	0.260	6	0.437	6	0.423	6	0.109	6
Saudi Arabia	0.372	3	0.427	5	0.623	3	0.576	2	0.067	5
Chile	0.157	7	0.145	7	0.390	7	0.376	7	0.033	7
India	0.155	8	0.061	8	0.343	8	0.323	8	0.029	8
Brazil	0.116	10	0.012	9	0.274	9	0.255	9	0.027	10
Canada	0.151	9	0.000	10	0.265	10	0.246	10	0.020	9

All the models used identical entropy weights $\left({\mathcal{W}}_{1}-{\mathcal{W}}_{8}\right)$ to ensure fairness in comparison; differences arise only from the ranking mechanisms.

The illustration in Fig. 6 provides a clear visualization of rank variations among green hydrogen-producing countries across different combined IVN-based MCDM models. The comparative analysis reveals that, as expected, minor ranking differences occur among methods such as IVN-Entropy-TOPSIS, IVN-Entropy-EDAS, IVN-Entropy-SAW, IVN-Entropy-WASPAS, and the proposed IVN-Entropy-NC-SIVNDM MARCOS model. This variation arises from differences in the computational structures of the models and the evaluation of multiple alternatives under eight quantitative criteria.

Table 15 presents the correlation matrix among the ranking outcomes of the IVN-based MCDM methods using common IVN-Entropy weights. Spearman’s rank correlation coefficients $\left(\rho =0.90-0.99\right)$ demonstrate a strong positive relationship and high consistency across all approaches. Minor variations in individual country rankings do not affect the overall trend, confirming that the proposed IVN-Entropy-NC-SIVNDM MARCOS model achieves excellent stability, robustness, and reliability in evaluating green hydrogen-producing countries.

**Table 15:** Spearman’s rank correlation $\left(\rho \right)$ among IVN-based MCDM methods using common IVN-Entropy weights

Methods	TOPSIS	EDAS	SAW	WASPAS	NC-SIVNDM MARCOS (Proposed)
TOPSIS	1	0.952	0.988	0.976	0.952
EDAS	0.952	1	0.964	0.927	0.976
SAW	0.988	0.964	1	0.988	0.939
WASPAS	0.976	0.927	0.988	1	0.903
NC-SIVNDM MARCOS (Proposed)	0.952	0.976	0.939	0.903	1

Overall, both sensitivity and comparative analyses validate the robustness, reliability, and generalizability of the proposed approach for evaluating green hydrogen performance.

Since the present study evaluates national-level green hydrogen readiness across multiple countries using aggregated macroeconomic, technological, and policy-oriented indicators, direct experimental or operational validation is not feasible. The analysis is inherently strategic and comparative, focusing on cross-country benchmarking rather than plant-level or real-time performance assessment. Accordingly, the robustness and reliability of the proposed IVN-Entropy-NC-SIVNDM MARCOS framework are validated through (i) comparative benchmarking with established interval-valued neutrosophic MCDM methods, assessing ranking consistency and discrimination capability, and (ii) extensive sensitivity analysis under multiple weight-variation scenarios to examine result stability. Such validation approaches are consistent with prior international country-level sustainability and energy policy assessment studies.

## Discussion

In this section, the implications of the obtained rankings are analyzed, encompassing implications for research and practice, and research limitations are discussed alongside the effectiveness, robustness, and interpretability of the proposed IVN-Entropy-NC-SIVNDM MARCOS framework in comparison with existing IVN-based MCDM approaches. Beyond robustness verification, it is also essential to highlight the methodological advantages and added value of the proposed framework compared with existing MARCOS-based and IVN-MCDM approaches.

### Advantages and added value of the proposed IVN-entropy-NC-SIVNDM MARCOS framework

The robustness analysis validates ranking stability across several weighting schemes and ranking methodologies; however, the proposed IVN-Entropy-NC-SIVNDM MARCOS framework offers distinct methodological advantages over previous approaches.

In contrast to classical and fuzzy MARCOS models, the proposed framework operates in an IVN context, enabling the concurrent modeling of uncertainty, indeterminacy, and inconsistency in green hydrogen data attributes that traditional fuzzy or intuitionistic fuzzy methods do not explicitly address.

The proposed NC-SIVNDM integrates both centroid and spread information from IVN numbers, thereby preserving uncertainty dispersion and preventing information loss associated with defuzzification-based measures. This results in enhanced discrimination, especially when alternatives demonstrate almost equivalent performance. The entropy-based objective weighting scheme offers a fully data-driven alternative to expert-based methods such as AHP, BWM, or FUCOM, thereby improving objectivity and reproducibility.

Thus, the integrated approach enhances interpretability and ranking reliability, rendering it appropriate for extensive, policy-focused benchmarking of national green hydrogen generation amid uncertainty. The methodological strengths also suggest significant implications for future research in sustainability-oriented decision-making.

### Implications for research

The current study advances the field of sustainable energy assessment by presenting the IVN-Entropy-NC-SIVNDM MARCOS framework, a hybrid model that objectively determines criterion weights and incorporates distance-based decision evaluation within the framework of IVN uncertainty. This methodological integration establishes a reproducible platform for subsequent MCDM investigations in uncertain, data-driven contexts characterized by expert prejudice and information indeterminacy. This finding shows that adding neutrosophic entropy to a distance-based MARCOS structure improves ranking stability and clarity. This is a good example for researchers developing more advanced decision-support systems for sustainability analytics.

This study scientifically validates the conceptual framework with real-world data from countries that produce green hydrogen, thereby extending the applicability of IVN-based methodologies beyond traditional industrial or supplier-selection scenarios. This research employs a data-centric methodology to measure performance across eight quantitative variables, including production capacity, electrolyser deployment, investment maturity, and efficiency metrics, thereby establishing a framework for future studies to conduct cross-sectoral sustainability comparisons.

The results also show that integrating objective entropy weighting with distance-based compromise ranking can be a useful approach to address difficult trade-offs among environmental, economic, and technological variables. Researchers in the future can build on this work by adding hybrid weighting systems (such as IVN-CRITIC-Entropy) or dynamic temporal datasets to examine how green hydrogen evolves over time. The suggested NC-SIVNDM distance formulation also opens new avenues for examining multidimensional uncertainty modeling in frameworks for evaluating green energy sources.

### Implications for practice

The results of this study provide substantial practical benefits for policymakers, investors, and stakeholders involved in developing the global green hydrogen sector. The proposed IVN-Entropy-NC-SIVNDM MARCOS framework offers a transparent and systematic method for evaluating countries' performance under uncertainty, aligning with prior MCDM-based research on sustainable hydrogen strategies and production assessments [86–88]. This model facilitates the identification of leading performers, the prioritization of investments, and the monitoring of progress toward national and international energy transition goals through a data-driven methodology.

The suggested framework integrates quantitative metrics, including production capacity, electrolyser advancement, cost efficiency, and investment readiness, to facilitate informed policy development and industrial decision-making. Authorities and energy organizations can use the findings to set standards for green hydrogen preparedness, develop incentive programs, and improve resource allocation for the implementation of large-scale projects. The framework offers crucial insights for private investors and industry strategists to pinpoint potential opportunities, assess technological maturity, and strengthen international collaborations in hydrogen production and export.

The present study establishes a practical basis for integrating objective, neutrosophic-based decision-support tools into sustainability evaluation systems. This approach promotes the adoption of structured assessment models that account for uncertainty, thereby improving transparency and consistency in decision-making processes. This contributes to the overall advancement of the global green hydrogen economy.

The country rankings indicate discernible variations in technology preparedness, investment maturity, and infrastructure development within the global green hydrogen sector, as seen from a policy and strategic perspective. Countries occupying higher ranks exhibit a harmonious integration of efficient low-emission hydrogen production, sophisticated electrolyser deployment, and concrete investment decisions, while lower-ranked nations primarily rely on declared capacities without corresponding implementation advances. These insights allow policymakers to differentiate between aspirational planning and actual performance, therefore facilitating evidence-based policy development and strategic prioritization in national hydrogen roadmaps.

From a contextual validation perspective, the obtained country rankings are broadly consistent with global patterns of green hydrogen leadership reported in international policy and investment assessments, particularly those documented in the IEA global hydrogen review. Countries ranked at the top of the proposed framework correspond to those identified by the IEA as frontrunners in electrolyser deployment, large-scale project announcements, and investment commitment, whereas lower-ranked countries are primarily characterized by early-stage planning or aspirational capacity targets. This alignment enhances the practical credibility of the proposed framework while acknowledging that the study does not claim causal or predictive validation.

## Research limitations

The present study introduced a hybrid MCDM framework by integrating the IVN-entropy weighting approach with the NC-SIVNDM MARCOS model to evaluate and rank green hydrogen–producing countries. While the proposed IVN-Entropy-NC-SIVNDM MARCOS method provides an objective, data-driven framework for managing uncertainty and improving ranking robustness, several limitations should be acknowledged.

First, the research relies primarily on secondary quantitative data obtained from international reports such as the global hydrogen review 2024. Consequently, the findings are limited by the completeness and reliability of the available datasets, which may not fully capture recent or region-specific developments in the hydrogen sector. Second, the analysis focuses exclusively on quantitative indicators and fails to integrate qualitative factors, such as policy frameworks, geopolitical risks, and social acceptance, which could further influence progress on green hydrogen.

Additionally, although the IVN environment effectively handles uncertainty and indeterminacy, it still relies on assumptions in transforming raw data into neutrosophic values, which may introduce minor computational approximations. Finally, the evaluation was conducted at the country level; hence, the results may not directly reflect intranational disparities in hydrogen production or infrastructure readiness.

Future studies may address these limitations by incorporating expert-based hybrid weighting schemes (e.g., IVN-CRITIC-Entropy), dynamic temporal datasets, and mixed quantitative–qualitative indicators to increase comprehensiveness. Expanding the application of the proposed framework to regional or sectoral analyses could also improve its generalizability and policy relevance.

## Conclusions

This study presented an innovative IVN-Entropy-NC-SIVNDM MARCOS methodology for assessing and ranking countries that produce green hydrogen amidst uncertainty. This work methodologically represents the inaugural effort to amalgamate the interval-valued neutrosophic (IVN) framework with a distance-based adaptation of the MARCOS approach. This innovation improves the management of uncertainty, indeterminacy, and reluctance in decision-making, providing greater transparency and reliability than conventional fuzzy or entropy-based models. The incorporation of IVN-Entropy for objective weighting and the NC-SIVNDM distance formulation enhances the ranking stability and methodological rigor, offering a novel avenue for neutrosophic MCDM research.

The empirical findings highlighted China, the European Union, and Australia as the leading countries in green hydrogen generation, demonstrating exceptional performance across production capacity, electrolyser deployment, investment maturity, and cost efficiency. The results illustrate the practical utility of the proposed model for consolidating quantitative indicators into a cohesive evaluation framework. This allows policymakers, energy planners, and investors to assess national hydrogen preparedness, inform investment priorities, and formulate evidence-based sustainability policies to promote the global hydrogen economy.

Comparative and correlation analyses with other IVN-based MCDM models, such as IVN-TOPSIS, IVN-EDAS, IVN-SAW, and IVN-WASPAS, confirm the stability and consistency of the proposed model. The strong correlation observed among these approaches confirms the reliability of the developed framework, positioning it as an effective decision-support tool for sustainability evaluation.

Notwithstanding these contributions, this study possesses specific limitations. The research is based on secondary data from the IEA global hydrogen review 2024, which may not adequately reflect regional disparities or transient operational fluctuations. Secondly, the assessment is static, representing present and declared capacities rather than their chronological progression. Ultimately, although the IVN framework adeptly addresses uncertainty and indeterminacy, converting quantitative indicators into interval-valued neutrosophic numbers may introduce abstraction and reduce granularity in certain circumstances.

The proposed IVN-Entropy-NC-SIVNDM MARCOS framework presents exciting opportunities for future exploration across various sustainability and decision-analysis fields. The ability to integrate uncertainty, indeterminacy, and objective weighting enables adaptability across diverse and intricate evaluation settings. This framework, in addition to assessing green hydrogen, can be applied to evaluate various renewable energy technologies, carbon–neutral infrastructure projects, circular economy initiatives, climate adaptation strategies, and smart manufacturing systems, particularly in contexts where multiple conflicting criteria and uncertain information are present. Furthermore, incorporating hybrid weighting methods, dynamic time-series data, and spatial or regional analytics may improve the model's accuracy in monitoring performance trends and facilitating real-time policy adjustments. Future investigations may also delve into its potential in financial sustainability evaluation, urban energy strategies, and transportation decarbonization, highlighting its interdisciplinary significance and strength in addressing high-impact, real-world decision-making challenges amid uncertainty.

## Data Availability

The dataset analyzed during the current study is publicly available in the *International Energy Agency (IEA) Global Hydrogen Review 2024 repository at: https:/www.iea.org/reports/global-hydrogen-review-2024.* All data used are secondary and openly accessible.
